# Dietary and socioeconomic risk factors for fumonisin exposure among women of reproductive age in 18 municipalities in Guatemala from 2013 to 2014

**DOI:** 10.1371/journal.pgph.0000337

**Published:** 2022-08-09

**Authors:** Ariel V. Garsow, Olga R. Torres, Jorge A. Matute, Ronald T. Riley, Julie R. Harris, Archana P. Lamichhane, Orion McCotter, Barbara B. Kowalcyk

**Affiliations:** 1 Department of Food Science and Technology, The Ohio State University, Columbus, OH, United States of America; 2 Laboratorio Diagnóstico Molecular, Guatemala City, Guatemala; 3 Centro De Investigación en Nutrición y Salud, Guatemala City, Guatemala; 4 Department of Environmental Health Science, University of Georgia, Athens, GA, United States of America; 5 Centers for Disease Control and Prevention, Atlanta, GA, United States of America; 6 RTI International, Research Triangle Park, NC, United States of America; 7 Translational Data Analytics Institute, The Ohio State University, Columbus, OH, United States of America; Mustafa Kemal University: Hatay Mustafa Kemal Universitesi, TURKEY

## Abstract

Fumonisin exposure is common in populations where maize is a dietary staple, such as in Guatemala, and has been associated with negative health outcomes including neural tube defects. The objective of this study was to estimate fumonisin B_1_ (FB_1_) exposure among Guatemalan reproductive-age women and develop a better understanding of the dietary and sociodemographic risk factors for exposure. A cross-sectional study in 18 municipalities in Guatemala was conducted. Midwives and study nurses enrolled consenting women and collected individual and household demographic and socioeconomic data. A food frequency questionnaire was administered to estimate quantity and types of food products consumed. A urine sample was collected and urinary fumonisin B_1_ (uFB_1_) concentration was measured. A univariable analysis was conducted to identify predictors of low/high uFB_1_. Multivariable logistic regression was used to calculate adjusted odds ratios (ORs) and 95% confidence intervals (CIs). In total, 775 women had analyzable urine samples. Higher uFB_1_ levels were associated with speaking Mayan (OR = 2.33, 95% CI:1.44–3.77), less than high school education (OR = 1.61, 95% CI:1.12–2.30), increasing dietary proportion of maize-based foods (OR = 1.02, 95% CI:1.01–1.03), and consumption of tostadas (fried tortillas) (OR = 1.11, 95% CI:1.02–1.22). Lower uFB_1_ levels were associated with consumption of highly processed maize-based foods (OR = 0.93, 95% CI:0.87–0.99). Tortillas were the most frequently consumed maize-based food among study participants and significantly associated with high uFB_1_ exposure in the univariable but not multivariable analysis. Consumption of >4,750 grams/week of maize-based foods, >5,184 g/week of locally produced maize-based foods, and >110 servings/week of tortillas were also significantly associated with high uFB_1_ exposure in univariable analysis. Populations with low socioeconomic status/education levels and high consumption of maize-based foods had higher fumonisin exposure. Interventions aimed at reducing the risk of exposure to mycotoxins through maize in Guatemala, including the increased consumption of non-maize-based foods, should be further explored.

## Introduction

Fumonisins are mycotoxins produced primarily by the fungus *Fusarium verticillioides*, a common contaminant of maize, and inhibit ceramide synthases [1–3]. Fumonisins are involved in the pathogenesis of numerous animal diseases [1, 4], are considered possibly carcinogenic in humans [1, 5], and have been associated with negative health outcomes including neural tube defects [4, 6–8]. Exposure to fumonisins occurs through consumption of contaminated food, predominantly maize and maize-based foods [1, 4]. People in low- and middle-income countries (LMICs) are disproportionately and continually exposed to higher quantities of fumonisins than persons in higher-income countries [4, 9], especially where diets comprise mostly maize, and practices and regulations to control fumonisins in the food supply are not easily implemented because most food is self-produced [1, 4]. While more than 20 fumonisin analogs have been identified, fumonisin B_1_ (FB_1_) is the predominant type in maize infected with *F*. *verticillioides*, accounting for approximately 70% of all forms of fumonisins found in maize.

Maize is a principal dietary component in Guatemala [10, 11]. Guatemalans whose diet is limited to maize-based foods are typically rural residents with a low socioeconomic level and less formal education [12]. Many Guatemalans have a diet low in variation, with maize as the primary source of calories [2, 11, 13]. For example, the diet of urban young Guatemalan adults, as described from a cohort study conducted in the Department of El Progreso, consists largely of maize tortillas, beans, sugar added to coffee, and highly processed foods like pizza and hamburgers in larger cities [14].

Given their diets and the agroecological conditions in Guatemala, Guatemalans are at risk of chronic exposure to fumonisins [1, 3, 13]. High levels of fumonisins have been found in maize grown at lower elevations where environmental conditions are conducive to both cultivation of maize, growth of fungi, and subsequent production of fumonisins [10, 11, 13, 15–17]. One study estimated FB_1_ intake in Guatemalan women (based on the levels of fumonisins found in the maize purchased from local markets) to be between 0.20–23 μg/ kg of body weight (bw) per day. Two additional studies were undertaken to estimate FB_1_ intake among 1,533 maize-consuming Guatemalan women from six communities representing historically high- and low-exposure regions of Guatemala [2, 11]. Results showed that urine from women in the three high-exposure communities had significantly higher levels of uFB_1_ (range: non-detected to 61.9 ng/ml) than urine from women in the three low-exposure communities (range: non-detected to 9.95 ng/ml; p <0.05). Urinary fumonisin B_2_ and fumonisin B_3_ were also detected at levels below that expected based on the level of contamination found in the maize used to prepare maize-based foods [2, 11]. In total, 11% of women in the low-exposure communities and 75% of women in the high-exposure communities had estimated total FB_1_ intakes that exceeded 2 μg/kg bw/day [2, 11], which is the provisional maximum tolerable daily intake (PMTDI) recommended by the World Health Organization (WHO) for FB_1_, FB_2_, and FB_3_ alone or in combination [1]. Studies in other high maize-consuming regions, including Mexico and parts of Sub-Saharan Africa, have demonstrated an association between consumption of maize-based foods and uFB_1_ levels [18–20]. In 2017, an evaluation by the Joint FAO/WHO Expert Committee on Food Additives (JECFA) concluded that uFB_1_ has been validated as a biomarker for FB_1_ intake in multiple human studies [1].

In low- and medium-income countries like Guatemala, regulations to control exposure to mycotoxins do not exist or are not enforced, and usually the analytical capacity to monitor the level of fumonisins in maize is not available [4]. Poor agricultural practices and a diet based on tortillas as staple food result in the possibility of high individual exposures, particularly among subsistence farmers and those dependent on maize sold for human consumption at the local markets [21].

Repeated consumption of fumonisin-contaminated maize has been identified as a potential risk factor for neural tube defects and stunting [22]. The objective of this study was to estimate FB_1_ exposure among Guatemalan reproductive-age women and to develop a better understanding of the dietary and sociodemographic risk factors for FB_1_ exposure among two departments in Guatemala with varying economic levels and high rates of neural tube defects [23]. The long-term goal is to use the identified risk factors to design future studies to better understand the possibility of fumonisins as a contributing factor in human disease in Guatemala. It was hypothesized that consuming a greater amount of maize and maize-based foods, mainly tortillas, and lower socioeconomic status are associated with higher uFB_1_ concentrations among Guatemalan women of reproductive age.

## Methods

### Ethics statement

The study had ethical approval number 9–13 of the National IRB of the Ministry of Health of Guatemala, and of INCAP Ethics Institutional Committee (CIE by Spanish Acronym) CIE-REV-030-2013. INCAP’s CIE has Federal Wide Assurance and acted as local IRB for Centers for Disease Control and Prevention (CDC); the field workers were trained to comply with all of both IRB’s recommendations. The researchers met all the requirements of both committees. Formal written informed consent or a fingerprint demonstrating consent was obtained by the midwives from the prospective participants. Study participants received a refreshment (a muffin) and Q50 (approximately $6.67 USD) for transportation expenses. If an individual was under 18 years of age, formal written informed consent or a fingerprint demonstrating consent was obtained from a parent or guardian and formal written assent or a fingerprint demonstrating assent was obtained from the individual. Additionally, since midwives were volunteers in the community, written informed consent was obtained from midwives as requested by the IRB at the Institute of Nutrition of Central America and Panama (INCAP).

The target population for this cross-sectional study was reproductive age women (aged 15–49 years) in two of the 22 departments (first-level geopolitical administrative areas in Guatemala) of the Republic of Guatemala, the department of Guatemala and the department of Alta Verapaz. Both departments comprise 17 municipalities (second-level geopolitical administrative areas in Guatemala). Women were recruited from all 17 municipalities in the department of Guatemala and, Cobán, from the department of Alta Verapaz, for a total of 18 study sites.

Women were recruited from March 2013 to January 2014 through a network of 246 midwives, two study nurses, and five field workers. Midwives are registered with the public health services of the county and provide healthcare services for persons who cannot afford private health services. As volunteers of the community health areas and respected members of the community, the midwives identified potential study participants and were paid Q50 to cover transportation expenses. Demographic data were collected from each study participant by hired data collectors, including municipality of residence and maternal age. Due to prior victimization and potential threats of violence in the areas that the survey was conducted [24], it was decided that asking subjects questions about household income could discourage subjects from participating in the survey. As such, indicators of socioeconomic status were collected from each study participant as a proxy for household income, including number of persons living in the household (1 to 7 or 8 to 18), educational level (above or below high school), and paid employment (housewife or paid worker) [25–27]. Low literacy rates are common among Guatemalan women; hence, face-to-face interviews were conducted by trained nurses/field workers who speak Spanish or Spanish-Q‘eqchi (Mayan dialect of Cobán). When a participant spoke another dialect, the interview was conducted with the help of local translators.

Women were also asked about their consumption of 61 food items representing 19 food groups (S1 Table) in the previous week using a food frequency questionnaire (FFQ) used extensively in similar studies [2, 11, 15, 28]. Data was anonymized at the time of collection.

Food groups were determined using the food composition table published by the INCAP. Units of consumption were reported to estimate the grams ingested (e.g.: one tortilla, one glass, one cup, one tablespoon, one piece of fruit, one tamal, one bag of tortrix, etc.) with each unit having a known average weight (S1 Table) [17]. Maize-based foods were categorized as locally produced (not highly processed), industrially produced highly processed (most likely do not contain fumonisins), or micronutrient-fortified (potentially contains fumonisins) to explore the role of processing conditions on fumonisin exposure. For example, industrially produced foods, such as those based on cornstarch (like atol de maicena), are subjected to extrusion during processing, and therefore have a low probability of containing fumonisins (S1 Table) [29]. The number and total quantity of reported foods consumed, the number and amount of reported maize-based foods consumed, and the percentage of total reported food consumption that was maize-based were estimated [17].

Individual exposure to FB_1_ was estimated using uFB_1_ (ng/ml) levels. A spot urine sample was collected from each woman and shipped frozen to the Laboratorio Diagnóstico Molecular in Guatemala City, where uFB_1_ was extracted and sent to USDA’s Russell Research Center in Athens, Georgia to be analyzed following published protocols [2, 28, 29]. Briefly, urine samples (9 ml) were adjusted to 10% acetonitrile using acetonitrile containing 1% formic acid. A total of 40 ng of U-[^13^C_34_]-FB_1_ (Sigma-Aldrich Corp., St. Louis, MO, USA) was added to every urine sample as an internal standard to allow for quantification of uFB_1_ levels in samples after extraction. Fumonisins were isolated on C_18_ solid phase extraction (SPE) cartridges (Sep-Pak R Classic C18 cartridges, Waters Corporation, Milford MA, USA). The loaded solid phase extraction cartridges were shipped to and eluted at USDA’s Russell Research Center in Athens, Georgia as described in Riley et al. [29] using 2 ml of 70% acetonitrile:30% water made to 0.1% formic acid. Separation of the eluates from the SPE cartridges was accomplished by reverse phase HPLC analysis conducted using a Finnigan Micro AS auto sampler coupled to a Surveyor MS pump (ThermoFisher, Woodstock, GA, USA). Separation was accomplished using an Imtakt Cadenza CW-C18 3μ particle size, 150 mm x 2 mm column (Imtakt USA, Philadelphia, PA, USA). The column effluent was directly coupled to a Finnigan LTQ-XL linear ion trap mass spectrometer (ThermoFisher, Woodstock, GA, USA) operated in the electrospray ionization positive ion mode. A complete set of pure standards (FB_1_, U-[^13^C_34_]-FB_1_, FB_2_, and FB_3_ at 1, 10, and 100 pg/μL) was run daily. Additionally, a 10 pg/μL standard was run at the beginning and end of each set of samples. Quantification of FB_1_ was based on internal standardization by the comparison of the areas under the chromatographic peaks for FB_1_ to the area of the known amount of U-[^13^C_34_]-FB_1_ added to the samples before processing. Results were expressed as ng FB_1_/ml of urine and the limit of detection was 0.07 ng/ml [29]. Urine samples were only analyzed for uFB_1_ levels. Sample analysis was conducted between 2013–2014.

Descriptive statistics were calculated for demographic, socioeconomic, dietary variables overall, by municipality, and by FB_1_ exposure group. Since distributions were right-skewed, nonparametric tests were used to assess differences between groups. Differences in distributions of categorical variables (municipality, language, number of persons living in the household, occupation, age group) were evaluated using chi-square tests. Differences in means of continuous variables (maternal age, uFB_1_ level, total grams and servings consumed, total grams and servings by food type, percent of total food consumed that is maize based) were evaluated using the Kruskal-Wallis test.

Univariable logistic regression was used to assess the relationship between FB_1_ exposure group and sociodemographics; total number of reported food types consumed; reported consumption of each food type; the reported number of servings of food type consumed; the reported amount of total food consumed in grams; the reported number of maize-based food items consumed; the reported amount of maize-based food consumed in grams; and the reported percentage of food consumed that was maize based. Odds ratios (ORs) and 95% confidence intervals (CIs) for food consumption variables reflect the change in odds of FB_1_ exposure with a one serving increase in food intake. Variables significant at α = 0.20 were then included in the multivariable analysis.

Multivariable logistic regression was used to calculate adjusted ORs and 95% CIs for comparing none/low and high FB_1_ exposure groups. The multivariable model was fit using stepwise selection and considered the following variables: sociodemographics; total number of reported food types consumed; reported consumption of each food type; the reported number of servings of food type consumed; the reported amount of total food consumed in grams; the reported number of maize-based food items consumed; the reported amount of maize-based food consumed in grams; and the reported percentage of food consumed that was maize based. All possible interactions between main effects were tested but none were significant and, thus, were excluded from the final model.

Sensitivity analyses were conducted to evaluate the impact of imputing missing food consumption data and the cut-off value for assigning high and low FB_1_-exposure groups. Imputing missing food consumption data as zero and the median amount consumed produced similar results (not presented).

To further explore the impact of reported consumption of maize-based foods on exposure, the analysis was re-run for high and low consumers of maize-based food groups and individual foods. For each food group and food item, the cut-off for high- and low-exposure groups was defined to be the mid-point of the range for the specific food group or item. Study participants were categorized as high or low consumers and the univariable analysis was repeated for each food group or item. These methods are similar to those conducted in previous research by members of the research team [15]. In brief, 1,100 randomly selected adults from the 22 departments of Guatemala (50 per department) provided information on the amounts of maize-based foods eaten during the previous 7 days. Those in the 60^th^ percentile of the distribution or higher were identified as high consumers of maize-based foods and consumed 4,750 grams/week. Those below the 60^th^ percentile were considered low consumers of maize-based foods [30].

Data were recorded by double entry and validation using Epi-Info 6.04d, and all statistical analyses were performed using SAS v.9.4 for Windows (SAS Institute Inc., USA). Ethical approval was obtained from Comité Nacional de Etica from the Ministry of Health of Guatemala, the Hospital General San Juan de Dios, Hospital Roosevelt, Area de Salud de Alta Verapaz, and the United States Centers for Disease Control and Prevention, through the Institutional Review Board (IRB) of INCAP. A final report of the project that included a description of the JECFA PMTDI levels was shared with local health authorities in the departments of Guatemala and Alta Verapaz. Additionally, several workshops about study findings, fumonisins in general and the risk of chronic exposure were conducted for health authorities, agricultural sector authorities, industry delegates, and the press. Additional information regarding the ethical, cultural, and scientific considerations specific to inclusivity in global research is included in the Supporting Information (S1 Checklist).

## Results

A total of 802 reproductive-aged women were recruited to participate in the study. Of these, 27 (3.37%) women had spot urine samples that were unanalyzable and were excluded from the analysis. Most of the participants spoke Spanish; consumed ≤4,750 g of maize-based food/week (n = 670, 86.45%); had uFB_1_ levels <0.5 ng/ml (532, 68.65%); were younger than 25 years old; had less than a high school education; had 2–7 persons living in their households; and were housewives (Table 1, S3 Table).

**Table 1:** Sociodemographic characteristics of study participants.

Characteristic		Overall (N = 775)	
		N	(%)
Age group			
	Less than 25 years	397	(51.23)
	25 to 29 years	147	(18.97)
	30 to 34 years	125	(16.13)
	35 years and above	106	(13.68)
**Language**			
	Spanish	661	(85.29)
	Mayan	105	(13.55)
	Unknown	9	(1.16)
**Education**			
	Less than high school	428	(55.23)
	High school and above	344	(44.39)
	Unknown	3	(0.39)
**Occupation**			
	Housewife	620	(80.00)
	Paid worker	153	(19.74)
	Unknown	2	(0.26)
**Number persons in household**			
	2 to 7 persons	710	(91.61)
	8 to 18 persons	64	(8.26)
	Unknown	1	(0.13)

**Fig 1:**
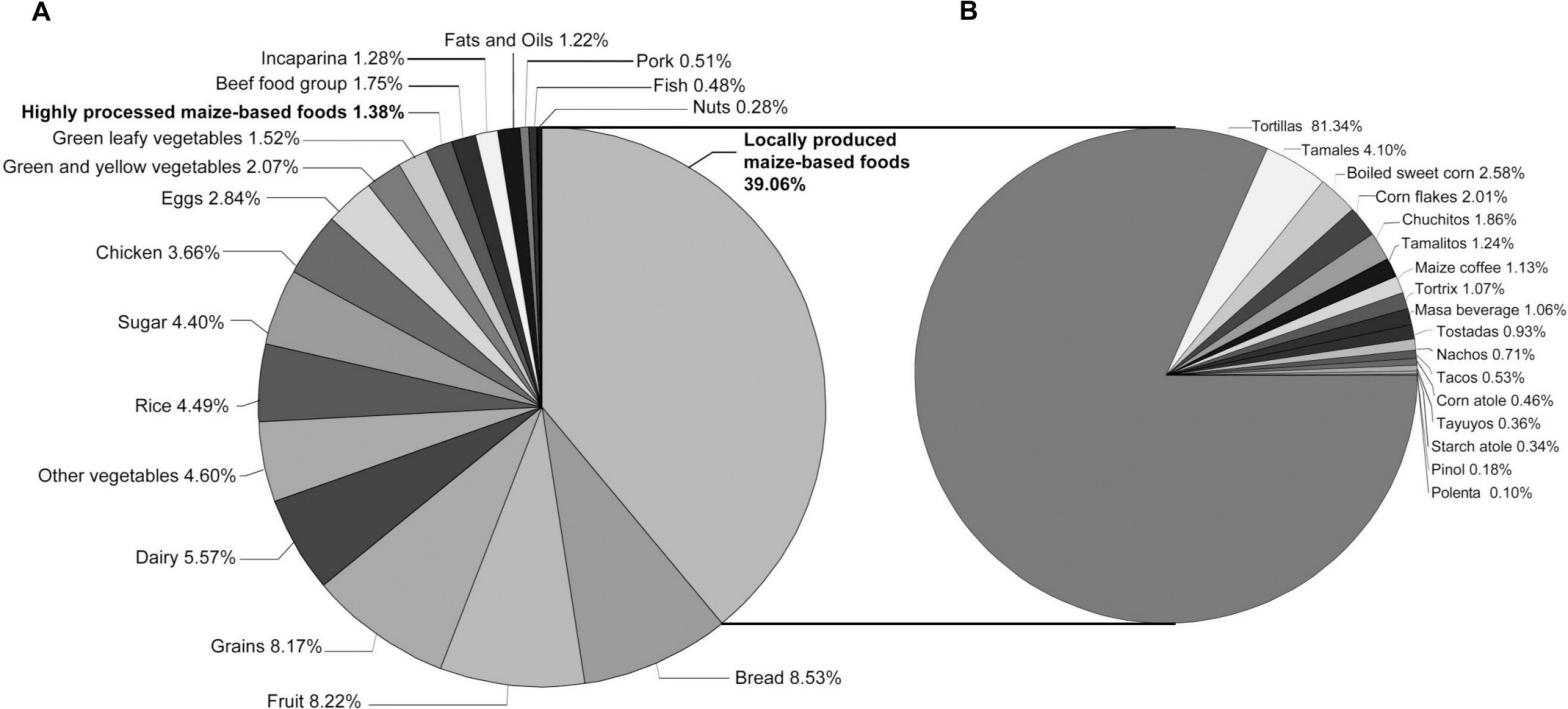
**A** Distribution of food groups, and **B**, highly processed and locally produced maize-based foods consumed in the week prior to study enrollment.

Overall, study participants consumed a median of 6.65 kg of food in the week prior to study enrollment (Table 2). Locally produced maize-based foods constituted 39.06% of all foods reported being consumed during the previous seven days and tortillas constituted 81.33% of all locally produced maize-based foods consumed (Fig 1).

**Table 2:** Total estimated weekly food consumption (kg), mean, median and range of number of servings consumed for selected food groups and maize-based food items per week overall and by uFB_1_ level (ng/ml)^1^*.

	Overall				Low (<0.5 ng/ml)				High (≥0.5 ng/ml)				
	N	Mean ± Std	Median	Range	N	Mean ± Std	Median	Range	N	Mean ± Std	Median	Range	P-value
uFB_1_ level	775	0.72 ± 2.03	0.10	0.00–32.44	532	0.08 ± 0.15	0.00	0.00–0.50	243	2.13 ± 3.20	1.28	0.51–32.44	.
Total food consumed^2^	749	7.41 ± 4.04	6.65	1.53–55.51	511	7.28 ± 4.01	6.46	1.53–55.51	238	7.70 ± 4.10	6.85	2.83–31.85	0.048
Locally produced maize-based foods (servings)^3^	767	67.14 ± 39.62	63.00	1.00–233.00	524	61.65 ± 37.94	57.00	1.00–233.00	243	78.98 ± 40.65	72.00	4.00–227.67	<0.001
Tortillas servings (1 serving = 40.00 g)	773	58.70 ± 36.22	56.00	0.00–220.00	530	53.77 ± 34.71	49.00	0.00–210.00	243	69.46 ± 37.17	63.00	3.00–220.00	<0.001
Tayuyos servings (1 serving = 55.00 g)	775	0.22 ± 1.08	0.00	0.00–14.00	532	0.13 ± 0.85	0.00	0.00–14.00	243	0.42 ± 1.44	0.00	0.00–9.00	0.003
Corn flakes servings (1 serving = 45.00 g)	773	1.25 ± 2.28	0.00	0.00–14.00	530	1.41 ± 2.43	0.00	0.00–14.00	243	0.92 ± 1.87	0.00	0.00–7.00	0.003

^1^ A complete list of all foods consumed, including maize-based foods, and their p values by uFB_1_ level is in S3 Table

^2^The total kilograms of all food consumed

^3^See S1 Table*p < 0.1, Kruskal-Wallis

**Fig 2:**
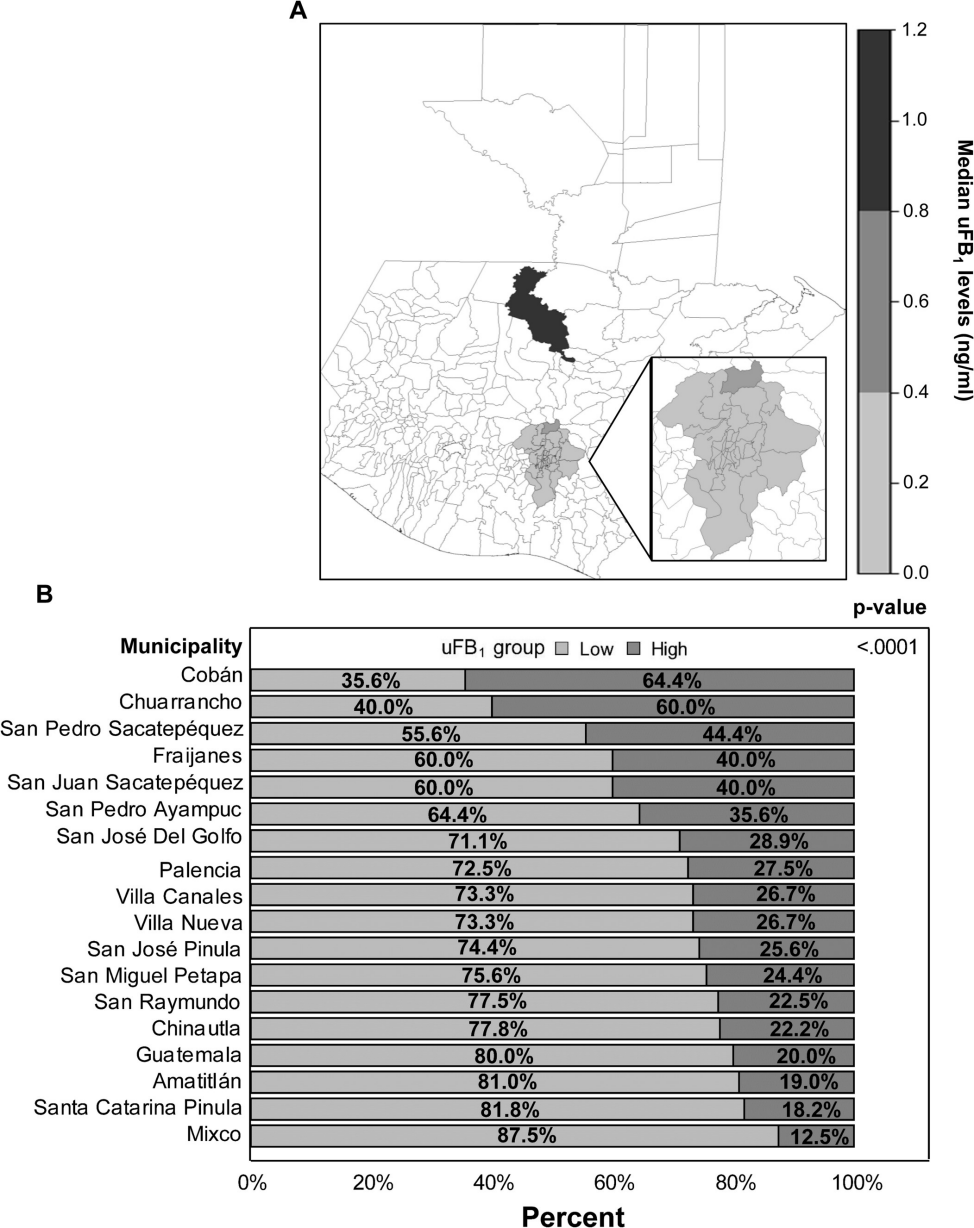
**A** Geographic distribution of median uFB_1_ groups in sampled municipalities. Grey shaded areas indicate research sites with scaled grey representing the relative uFB_1_ groups. The darkest area is the municipality of Cobán in the department of Alta Verapaz; all other grey shaded areas are municipalities in the department of Guatemala and white areas were not sampled in this study. p-value calculated using chi-square test. **B** Percentage of the study participants classified as low (<0.5 ng/ml) or high (≥0.5 ng/ml) uFB_1_ groups by municipality.

Of the 775 women included in the analysis, 398 (51.35%) had detectable uFB_1_ levels and 243 (31.35%) had levels that exceeded 0.5 ng/ml and were classified as having high exposure (Table 2). Since levels of uFB_1_ were not expected to be uniform across the population, FB_1_ exposure was categorized as a dichotomous variable: low/no exposure (uFB_1_ < 0.5 ng/ml) and high exposure (uFB_1_ ≥ 0.5 ng/ml). The cut-off point of 0.5 ng/ml for high and low exposure was chosen because, in previous studies [2, 11, 28], a uFB_1_ level of 0.5 ng/ml was estimated to be approximately equal to the JECFA PMTDI of 2 μg total FB/kg bw/day [1]. Specifically, an uFB_1_ concentration of 0.5 ng/ml is estimated to reflect an FB_1_ intake of 1.67 μg/kg bw/day [1, 2], assuming that the average woman weighs 60 kg, has a daily urine output of 1,000ml, and 0.5% of total FB_1_ intake is excreted [28]. Assuming FB_1_ comprises 70% of total FB (fumonisin) exposure, this suggests 0.5 ng/ml of FB_1_ in the urine is likely to indicate a total FB intake >2ug/kg bw/day, which is the current JECFA PMTDI above which there is a higher risk for adverse health outcomes associated with consumption of fumonisins [1].The overall mean uFB_1_ level was 0.72 ± 2.03 ng/ml, with the low-exposure group having a mean of 0.08 ± 0.15 ng/ml and the high-exposure group having a mean of 2.13 ± 3.20 ng/ml (Table 2, Fig 2A and 2B). Cobán (64.44%) and Chuarrancho (60.00%) were the two municipalities with the highest percentages of high-exposure women, while Mixco (12.50%) and Santa Catarina Pinula (18.18%) had the lowest percentages (Fig 2, S2 Table).

**Fig 3:**
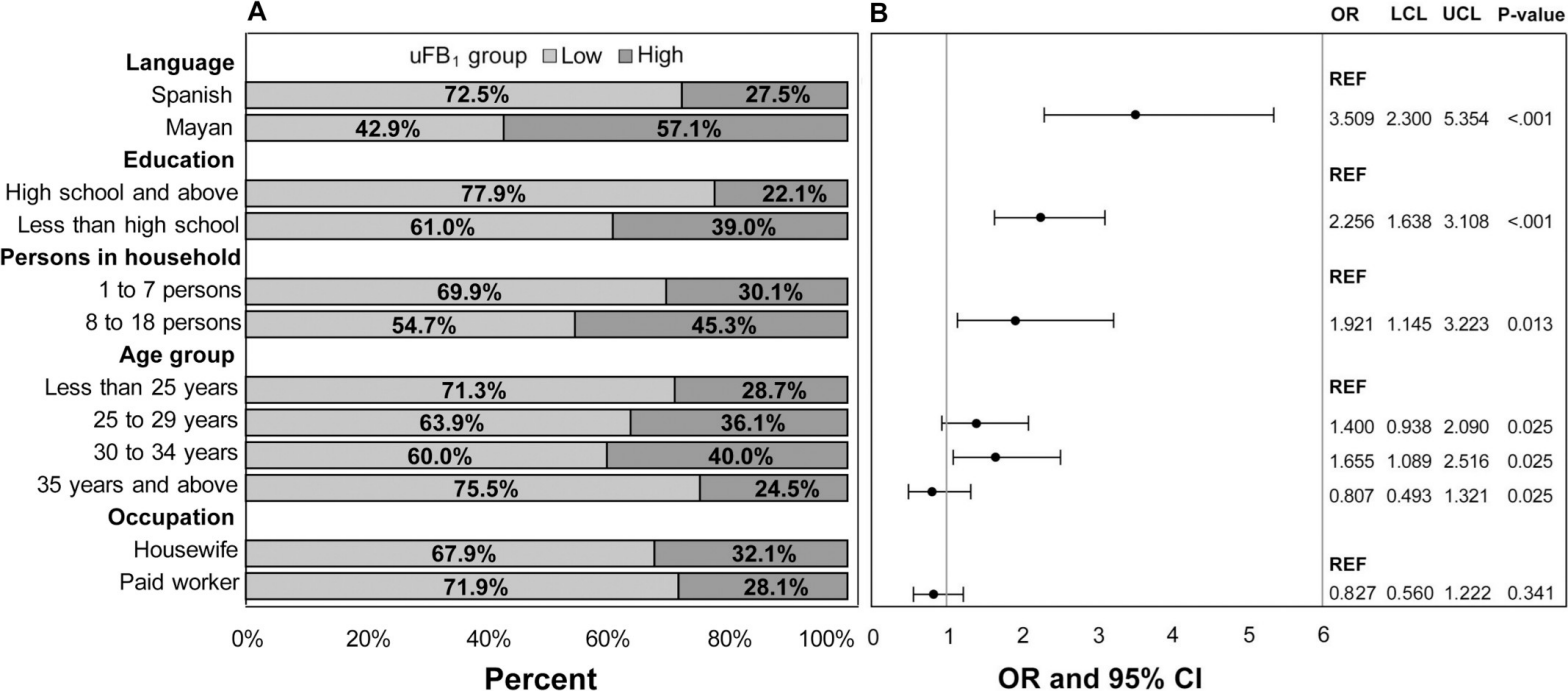
**A** Sociodemographic characteristics by low (<0.5 ng/ml) or high (≥0.5 ng/ml) uFB_1_ groups. **B** Univariable results for sociodemographic characteristics, including odds ratios (OR), upper (UCL) and lower (LCL) 95% confidence levels, and p-values from logistic regression.

**Fig 4:**
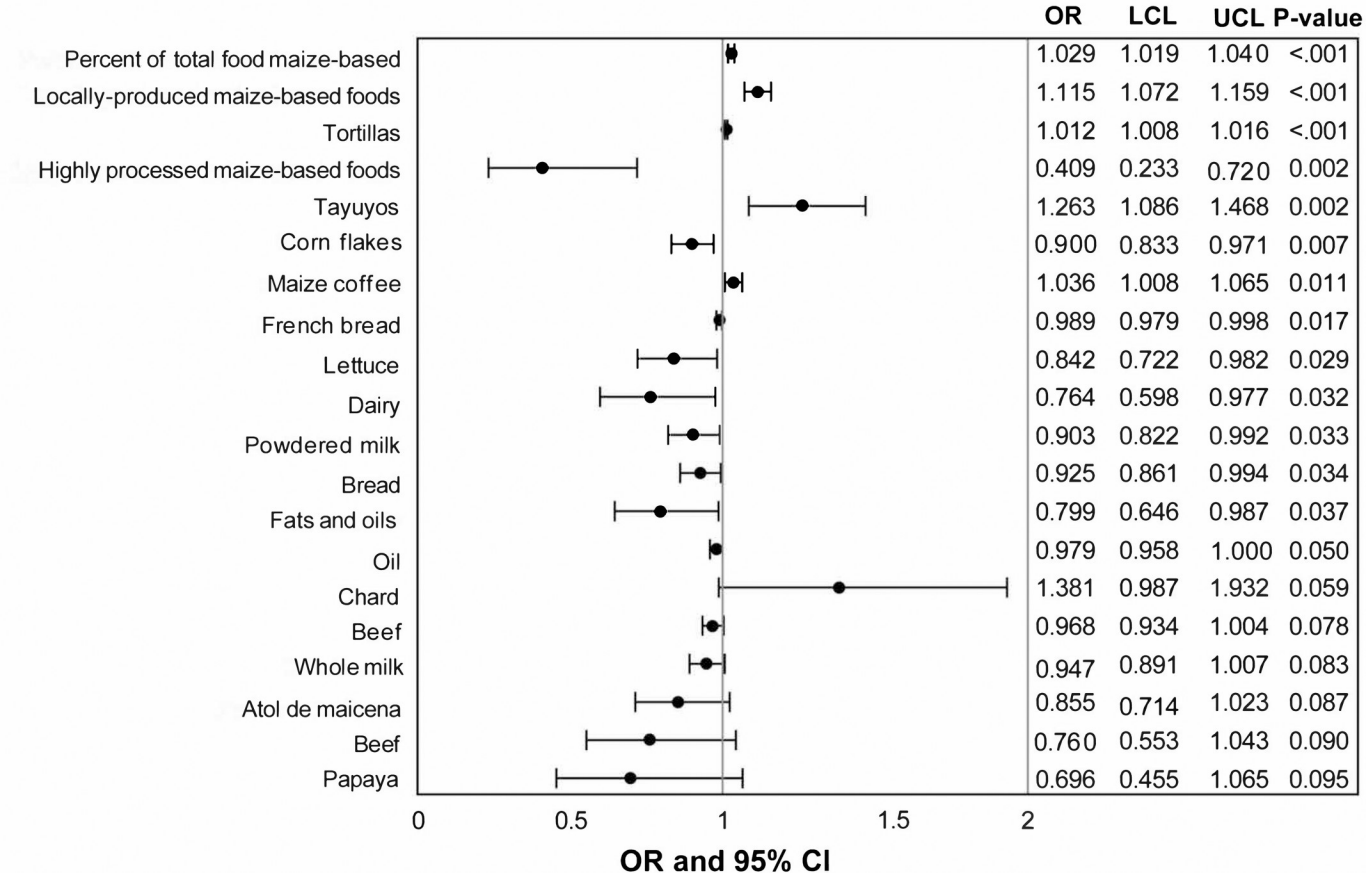
Dietary factors associated with uFB_1_ exposure group in univariable analysis. Unit is servings unless otherwise noted. Odds ratios (OR), upper (UCL) and lower (LCL) 95% confidence levels, and p-values from logistic regression.

In the univariate analysis, women with high uFB_1_ levels were more likely to speak Mayan; be less educated; have more persons living in the household; and be 30–34 years of age compared to reproductive-aged women with no/low uFB_1_ levels (Fig 3A and 3B). Women in the high-exposure group consumed significantly more maize-based foods than women in the low-exposure group (Fig 3B, S3–S5 Tables). Additionally, the low-exposure group ate a median of 49 tortillas per week while the high-exposure group ate a median of 63 tortillas per week (Table 2). The percent of total foods consumed that were maize-based and consumption of locally-produced maize-based foods, tortillas, and tayuyos (thick tortillas filled with beans) were significantly associated with high exposure (Fig 4). Reported consumption of highly processed maize-based foods, corn flakes, French bread, lettuce, dairy, powdered milk, the fats and oils food group, and oil as a food item were significantly associated with low exposure. Reported consumption of chard was marginally associated with high exposure while reported consumption of beef, whole milk, atol de maicena (a thick beverage made of starch), beef, and papaya were marginally associated with low exposure. Significant differences were also observed for language spoken (Spanish versus Mayan), reported maize consumption (≤ or > 4,750 g/week), uFB_1_ levels (< or ≥ 0.5 g/ml), age group, education (high school and above or less than high school), occupation (housewife or paid worker), and number of persons in household (1 to 7 or 8 to 18) when comparisons were made between municipalities using a Kruskal-Wallis test (S2 Table).

**Fig 5:**
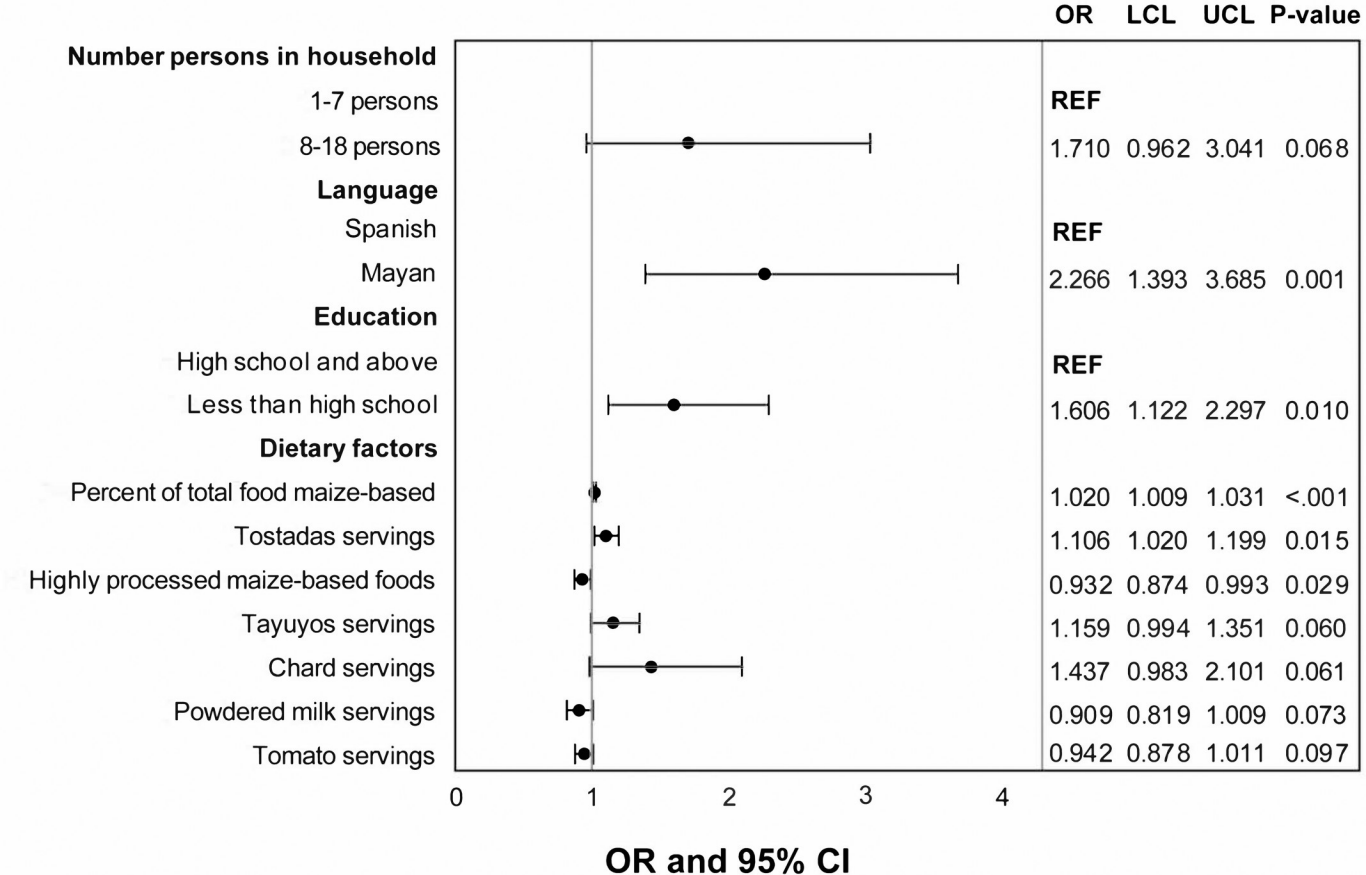
Significant demographic and dietary factors significantly associated with uFB_1_ exposure group in multivariable analysis (p<0.1). Odds ratios (OR), upper (UCL) and lower (LCL) 95% confidence levels, and p-values from logistic regression.

In the multivariable analysis, the percent of total food consumed that was maize-based, language spoken, education level, reported consumption of tostadas, and reported consumption of highly processed maize-based foods were significantly associated with exposure group (Fig 5). For every 1% increase in percentage of total food consumed that was maize-based, the odds of having high uFB_1_ levels (>0.5 ng/ml) increased by 1.03 (95% CI: 1.02–1.04). Those who spoke a Mayan language had increased odds of having high uFB_1_ levels compared to those who spoke Spanish. Women with less than a high school education had significantly higher odds of having high uFB_1_ levels than those with more than a high school education. One serving increases in tostadas, which are fried tortillas, were significantly associated with increased odds of high uFB_1_ levels. One serving increases in highly processed maize-based foods were significantly associated with decreased odds of high uFB_1_ levels. The number of persons living in a household, reported consumption of tayuyos or chard were marginally associated with high exposure, while reported consumption of powdered milk or tomatoes were marginally associated with low exposure. The final multivariable logistic regression model had a significant fit (p < 0.001) and did not violate the Hosmer-Lemeshow goodness of fit test (p > 0.1008).

**Fig 6:**
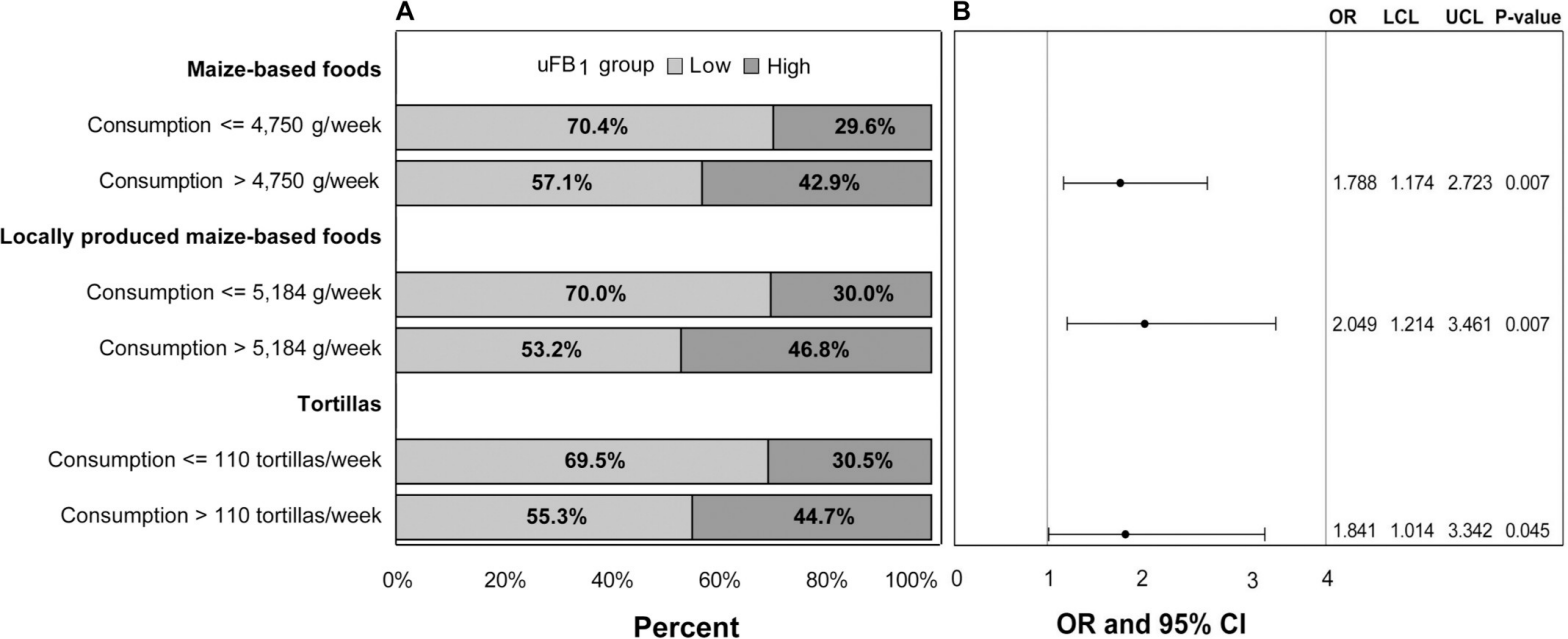
**A** Significant maize-based food groups and foods associated with low (<0.5 ng/ml) or high (≥0.5 ng/ml) uFB_1_ groups in categorical analysis. (**B**) Odds ratios (OR), upper (UCL) and lower (LCL) 95% confidence levels, and p-values from logistic regression.

In the categorical analysis, high reported consumption of maize-based foods, locally-produced maize-based foods, and tortillas were significantly associated with high exposure to uFB_1_ (Fig 6). Reported consumption of more than 110 servings/week of tortillas increased the odds of having high exposure to uFB_1_ by 1.84 (95% CI: 1.17–2.84).

The study was designed to estimate the prevalence of FB_1_ exposure while maximizing geographic diversity, but no formal sample size calculations were performed; rather, the sample size was driven by budgetary and laboratory constraints. A retrospective power analysis showed that a sample size of 775 provided >99% power to detect an OR of 1.09 at a significance level of 0.05, assuming a response rate of 31.35% for high FB_1_ exposure.

## Discussion

Nearly a third of all women and two-thirds of women in Cobán had uFB_1_ levels that exceeded 0.5 ng/ml. This level of uFB_1_ has been estimated to indicate a total FB_1_ intake that approximates or exceeds the JECFA PMTDI of 2 μg/kg bw/day. The JECFA PMTDI of 2 μg/kg bw/day was derived using dose-response animal toxicity studies (incorporating an uncertainty factor of 100 for intra- and interspecies variation) [1]. Elevation of sphingolipid metabolites in blood spots from Guatemalan women, indicative of FB_1_ inhibition of ceramide synthase, have been shown to first occur in the window of uFB_1_ concentration that was greater than 0.5 but less than 1.0 ng/ml [2]. The evidence suggests that fumonisin inhibition of ceramide synthase is the proximate cause of animal diseases associated with exposure to fumonisins [1, 2, 31]. These data (along with previous studies) provide an estimate of exposure of FB_1_ in women in Guatemala which can be used to better understand the possible adverse health effects of fumonisin on the population.

Reported consumption of maize-based foods was significantly associated with higher uFB_1_ levels. Tortillas comprise more than 80% of the Guatemalan diet and are likely one of the principal sources of fumonisin exposure in Guatemala [10, 11, 15, 19]. Reported consumption of maize-based foods was significantly associated with higher fumonisin exposure based on uFB_1_ levels. As suspected, processing seemed to reduce exposure. For example, reported consumption of locally processed maize-based foods, such as tortillas and other tortilla-based dishes (tostadas and tayuyos) was associated with higher FB_1_ exposure, while reported consumption of highly processed foods that are unlikely to contain fumonisins, such as corn flakes, was associated with lower exposure levels. Many highly processed maize-based foods are prepared with nixtamalized maize. Nixtamalization has been found to reduce some forms of fumonisins in the original maize [32]. However, if fumonisin levels are high in maize, the reduction of fumonisins that occurs through nixtamalization may still result in high levels of contamination in the nixtamalized maize [32, 33]. Due to the widespread consumption of tortillas, the ability to detect differences in the quality of maize used to produce tortillas may have been confounded. The marginal association of higher uFB_1_ exposure in both the univariable and multivariable analysis with reported chard consumption demonstrates this confounding as chard is almost exclusively eaten with tortillas in Guatemala. Additional research is needed to further elucidate the role of tortilla consumption in mycotoxin exposure.

These results support the conclusion that communities with high uFB_1_ are also likely to have high contamination in the maize sold for preparing maize-based food for human consumption. Two studies in Guatemala have shown that the calculated fumonisin intake based on either the uFB_1_ levels in spot urine samples or the total fumonisin in the maize collected at the same time from local markets were correlated [2, 11]. Communities with high uFB_1_ levels (Jutiapa, Chiquimula, and Santa Rosa) had higher contamination in the maize sold for preparing maize-based food for human consumption compared to communities with low uFB_1_ levels (Chimaltenango, Escuintla, Sacatepéquez). This finding is also supported by the results of a study of the kinetics of urinary FB_1_ excretion in humans consuming maize-based diets where uFB_1_ increased while consuming the FB-contaminated diets and quickly became non-detectable when consumption of the FB_-_contaminated diets ceased [28].

Results from this study suggest that reproductive-aged women in Guatemala consume substantial amounts of maize-based foods and increased reported consumption of these foods was associated with higher uFB_1_ levels. Additionally, consumption of multiple types of foods—such as lettuce, dairy, powdered milk, bread, fats and oils—were associated with lower uFB_1_ levels. In a randomized controlled trial conducted in 2019 that examined the variation in diet of 240 Guatemalan pregnant women, results indicated that 50% of the participants had diets that included consumption of staple foods (tortillas, bread, and rice) as well as meat, dairy (cheese), eggs, and pulses [34] Multiple studies have shown low diet variation and high maize-based food consumption in Guatemala [10, 11, 21]. Mendoza et al. [21] reported that nearly half of those interviewed in the department of Huehuetenango consumed more than 600 g of maize-derived foods/day, with some individuals ingesting as much as 3,000 g/day demonstrating the low diet variation of these populations [34].

Factors associated with poverty and indicative of lower socioeconomic status (i.e., education level, language spoken, number of persons in household, occupation) were significantly associated with high exposure to FB_1_. Specifically, higher uFB_1_ levels were observed in Mayan speakers, women living in crowded households, and women of lower education and lower socioeconomic levels. Lower socioeconomic status has been associated with higher reported maize consumption, a less diverse diet, and a potential for increased mycotoxin exposure [4]. The connection between FB_1_ exposure and poverty, lack of access to a variety of foods, and culture of high maize consumption need to be further understood to determine their relation to high levels of adverse health outcomes (such as stunting) on a population level in Guatemala. Additionally, research on socially and culturally appropriate interventions that reduce mycotoxin exposure to individuals by reducing maize contamination and increasing consumption of foods that are less likely to be contaminated are needed.

This study expands the knowledge of mycotoxin exposure in Guatemala and further demonstrates the utility of uFB_1_ as a biomarker to determine and monitor fumonisin intake. Previous research has demonstrated exposure to other mycotoxins (such as aflatoxins) through consumption of maize-based products. High levels of aflatoxin-albumin adducts in blood samples have been associated with high consumption of tortillas in Guatemala [35]. Additionally, in a cross-sectional study conducted in 2016, aflatoxin-lysine adducts (AFB_1_-Lys) were measured in the blood of 100% of study participants (443 adults ≥ 40 years old) living in five departments of Guatemala where the Institute of Cancer in Guatemala (INCAN) detected high rates of liver cancer with low prevalence of viral hepatitis antibodies [36]. Co-exposure to mycotoxins, like aflatoxin B_1_ (AFB_1_) and FB_1_, is a concern because FB_1_ is a proven tumor promoter of AFB_1_ liver carcinogenicity in animal models. The incidence of chronic liver disease and stunting are high where co-exposure to both mycotoxins are high [1]. As a result, the concluding recommendation of the 83^rd^ JECFA included reduction of exposure to these mycotoxins in LMICs [1]. Additional studies that estimate mycotoxin (e.g., fumonisin, aflatoxin) levels in maize-based foods throughout Guatemala can provide better insight into relationships between dietary consumption and mycotoxin exposure. Further studies should also be conducted to explore the association between mycotoxin exposure and adverse birth outcomes (e.g., low birth weight, neural tube defects, growth retardation, liver and renal disease) especially in the populations consuming food products highly contaminated with mycotoxins. The findings of this study can be used to inform the design of these studies and develop interventions that can effectively reduce exposure in countries that consume high amounts of foods that are likely to be contaminated, like Guatemala.

This study has several limitations that should be considered when interpreting the results. First, the FFQ asked about food consumption in the previous week, which may not be representative of the woman’s overall diet and could potentially bias results. FFQs estimate an individual’s usual food intake over a defined period, which is easier and more economical to conduct than dietary 24-hour recalls [2, 11, 37]. However, FFQs rely on reported consumption by the respondent, which introduces recall bias. Although, studies have shown that dietary patterns do not change much over time with a consistently high consumption of maize-based foods [10, 11, 28]. Further, the FFQ used in this study was developed in 2010 which could also introduce bias. However, studies have shown that dietary patterns have not changed since 2010 [12, 38, 39]. Calculating a dietary diversity score and total caloric intake was beyond the scope of this manuscript [40]. Due to the high consumption of maize-based foods in this population [10, 11, 28], the FFQ included the consumption of maize-based food items. Additionally, variables representing total servings consumed of 19 food groups and percent of maize-based foods were evaluated for inclusion in the multivariate model indirectly demonstrating dietary diversity and total energy intake in this population. Second, the study focused on only two regions within Guatemala, which weakens the extrapolation of the results to all reproductive-aged women in Guatemala. Third, household maize samples were not collected in this study, which would have provided supporting evidence of exposure estimates based on uFB_1_. Knowledge of both the amounts of maize-based foods consumed and the levels of FB_1_ contamination of the food are necessary to predict the levels of FB_1_ intake. In the absence of analysis of FB_1_ in the foods consumed, uFB_1_ serves as a biomarker for recent (24 to 48 h) FB_1_ intake [1, 2]. Finally, this study examined only one type of mycotoxins, fumonisins, even though maize can be contaminated with other mycotoxins such as aflatoxins. As noted previously, co-exposure to fumonisins and aflatoxins could significantly impact human health and should be the focus of future research.

This is the first cross-sectional study to explore the association between reported food consumption and FB_1_ exposure among Guatemalan women of reproductive age. Reproductive-aged women in Guatemala have uFB_1_ levels that indicate an exposure to FB_1_ that frequently exceeds the JECFA PMTDI. Results from this study suggest that there is an association between low socioeconomic status and/or education and high levels of reported consumption of maize-based foods (mainly tortillas) with FB_1_ exposure. Results of this study contribute to the body of evidence that consumption of locally processed maize products (e.g., home-cooked tortillas) is associated with higher exposure to mycotoxins.

## Supporting information

S1 Checklist Ethical, cultural, and scientific considerations specific to inclusivity in global research. (DOCX)

S1 Table Food groups and serving size (g) for associated food items. (DOCX)

S2 Table Maize consumption, uFB_1_ levels, and socio-demographic characteristics by municipality. (DOCX)

S3 Table Mean and median intakes (servings) of food items by FB_1_ exposure group. (DOCX)

S4 Table Consumption of food groups by department. (DOCX)

S5 Table Mean and median consumption of food groups by municipality. (DOCX)

S6 Table Data dictionary for raw data for article including variable names, description, and units. (CSV)

S7 Table Raw data for article. (CSV)
