# Supplementary material for: Dietary and socioeconomic risk factors for fumonisin exposure among women of reproductive age in 18 municipalities in Guatemala from 2013 to 2014
Source: PLOS Glob Public Health. 2022 Aug 9;2(8):e0000337. doi: 10.1371/journal.pgph.0000337 (PMC10021672; doi:10.1371/journal.pgph.0000337)
Supplement: S1 Table — (DOCX) [file pgph.0000337.s002.docx]

**S1 Table** Food groups and serving size (g) for associated food items.

| **Group Name** | **Food Items Included in Group** |
| --- | --- |
| Locally produced maize-based foods | Boiled corn on the cob (115.00 g), chuchitos (75.00 g), corn atole (sweet beverage) (40.00 g), maize coffee (20.00 g), masa beverage (40.00 g), nachos (60.00 g), pinol (28.75 g), polenta (corn flour) (28.5 g), tacos (35.00 g), tamales (200.00 g), tamalitos (55.00 g), tayuyos (55.00 g), tortillas (40.00 g), tostadas (25.00g) |
| Highly processed maize-based foods^1^ | Starch atole (40.00 g), corn flakes (45.00 g), tortrix (40.00 g) |
| Micronutrient fortified maize-based foods^1^ | Incaparina (36.00 g) |
| Nuts | Peanuts (28.75 g) |
| Grains | Beans (28.75 g), haba grano seco (28.75 g), haba flour (28.75 g), soy flour (28.75 g) |
| Rice | Rice (28.75 g) |
| Bread | French bread (25.00 g), sweet bread (20.00 g) |
| Milk, dairy | Cheese (28.75 g), cream (23.00 g), Whole milk (230.00 g), powdered milk (28.75 g) |
| Eggs | Eggs (47.00 g) |
| Beef | Beef (28.75 g), beef liver (28.75 g), beef kidney (28.75 g) |
| Pork | Pork (28.75 g) |
| Chicken | Chicken (28.75 g), chicken liver (28.75 g) |
| Fish | Fish (28.75 g) |
| Green leafy vegetables | Bledo (65.00 g), chard (65.00 g), lettuce (80.00 g), macuy (65.00 g), spinach (65.00 g), water cress (65.00 g) |
| Green and yellow vegetables | Asparagus (60.00 g), avocado (80.00 g), broccoli (65.00g g), green beans (65.00 g), green peas (65.00 g) |
| Other vegetables | Beet (80.00 g), cabbage (400.00 g), cauliflower (65.00 g), onion (45.00 g), tomato (70.00 g) |
| Fruit | Banana (110.00 g), orange (80.00 g), papaya (400.00 g), tangerine (60.00 g) |
| Fats and oils | Oil (12.00 g), lard (15.00 g) |
| Sugar | Sugar (7.00 g) |

^1^Processed so that fumonisin contamination is unlikely (purified starch, extrusion cooking, etc.)
