# Supplementary material for: Dietary and socioeconomic risk factors for fumonisin exposure among women of reproductive age in 18 municipalities in Guatemala from 2013 to 2014
Source: PLOS Glob Public Health. 2022 Aug 9;2(8):e0000337. doi: 10.1371/journal.pgph.0000337 (PMC10021672; doi:10.1371/journal.pgph.0000337)
Supplement: S2 Table — (DOCX) [file pgph.0000337.s003.docx]

**S2 Table.** Maize consumption, uFB_1_ levels, and socio-demographic characteristics by municipality.

| **Characteristic** | | **Overall**  **n= 775**  **Cases (%)** | | **Amatitlán**  **n= 42**  **Cases (%)** | | **Chinautla**  **n= 45**  **Cases (%)** | | **Chuarrancho**  **n= 45**  **Cases (%)** | **Cobán**  **n= 45**  **Cases (%)** | | **Fraijanes**  **n= 45**  **Cases (%)** | | **Guatemala**  **n= 40**  **Cases (%)** | | **Mixco**  **n= 40**  **Cases (%)** | |
| --- | --- | --- | --- | --- | --- | --- | --- | --- | --- | --- | --- | --- | --- | --- | --- | --- |
| Language | Unknown | 9 (1.16) | 0 (0.00) | | 1 (2.22) | | 0 (0.00) | | 0 (0.00) | 0 (0.00) | | 1 (2.50) | | 0 (0.00) | |  |
|  | Spanish | 661 (85.29) | 40 (95.24) | | 44 (97.78) | | 25 (55.56) | | 7 (15.56) | 41 (91.11) | | 35 (87.50) | | 36 (90.00) | |  |
|  | Mayan | 105 (13.55) | 2 (4.76) | | 0 (0.00) | | 20 (44.44) | | 38 (84.44) | 4 (8.89) | | 4 (10.00) | | 4 (10.00) | |  |
| Maize Consumption | ≤4750 g/week | 670 (86.45) | 39 (92.86) | | 42 (93.33) | | 37 (82.22) | | 23 (51.11) | 38 (84.44) | | 35 (87.50) | | 36 (90.00) | |  |
|  | >4750 g/week | 105 (13.55) | 3 (7.14) | | 3 (6.67) | | 8 (17.78) | | 22 (48.89) | 7 (15.56) | | 5 (12.50) | | 4 (10.00) | |  |
| uFB_1_ level | Low (<0.5 ng/ml) | 532 (68.65) | 34 (80.95) | | 35 (77.78) | | 18 (40.00) | | 16 (35.56) | 27 (60.00) | | 32 (80.00) | | 35 (87.50) | |  |
|  | High (≥0.5 ng/ml) | 243 (31.35) | 8 (19.05) | | 10 (22.22) | | 27 (60.00) | | 29 (64.44) | 18 (40.00) | | 8 (20.00) | | 5 (12.50) | |  |
| Age group | Less than 25 years | 397 (51.23) | 23 (54.76) | | 28 (62.22) | | 21 (46.67) | | 20 (44.44) | 29 (64.44) | | 22 (55.00) | | 25 (62.50) | |  |
|  | 25 to 29 years | 147 (18.97) | 10 (23.81) | | 3 (6.67) | | 12 (26.67) | | 15 (33.33) | 8 (17.78) | | 6 (15.00) | | 6 (15.00) | |  |
|  | 30 to 34 years | 125 (16.13) | 5 (11.90) | | 6 (13.33) | | 6 (13.33) | | 8 (17.78) | 5 (11.11) | | 6 (15.00) | | 8 (20.00) | |  |
|  | 35 years and above | 106 (13.68) | 4 (9.52) | | 8 (17.78) | | 6 (13.33) | | 2 (4.44) | 3 (6.67) | | 6 (15.00) | | 1 (2.50) | |  |
| Education | Unknown | 3 (0.39) | 0 (0.00) | | 0 (0.00) | | 1 (2.22) | | 0 (0.00) | 0 (0.00) | | 0 (0.00) | | 0 (0.00) | |  |
|  | Less than high school | 428 (55.23) | 21 (50.00) | | 20 (44.44) | | 34 (75.56) | | 40 (88.89) | 30 (66.67) | | 20 (50.00) | | 17 (42.50) | |  |
|  | High school and above | 344 (44.39) | 21 (50.00) | | 25 (55.56) | | 10 (22.22) | | 5 (11.11) | 15 (33.33) | | 20 (50.00) | | 23 (57.50) | |  |
| Occupation | Unknown | 2 (0.26) | 0 (0.00) | | 0 (0.00) | | 1 (2.22) | | 0 (0.00) | 0 (0.00) | | 1 (2.50) | | 0 (0.00) | |  |
|  | Housewife | 620 (80.00) | 35 (83.33) | | 39 (86.67) | | 26 (57.78) | | 44 (97.78) | 38 (84.44) | | 26 (65.00) | | 26 (65.00) | |  |
|  | Paid worker | 153 (19.74) | 7 (16.67) | | 6 (13.33) | | 18 (40.00) | | 1 (2.22) | 7 (15.56) | | 13 (32.50) | | 14 (35.00) | |  |
| Number persons in household | Unknown | 1 (0.13) | 0 (0.00) | | 0 (0.00) | | 0 (0.00) | | 0 (0.00) | 0 (0.00) | | 0 (0.00) | | 0 (0.00) | |  |
|  | 1 to 7 persons | 710 (91.61) | 39 (92.86) | | 38 (84.44) | | 42 (93.33) | | 38 (84.44) | 45 (100.00) | | 39 (97.50) | | 38 (95.00) | |  |
|  | 8 to 18 persons | 64 (8.26) | 3 (7.14) | | 7 (15.56) | | 3 (6.67) | | 7 (15.56) | 0 (0.00) | | 1 (2.50) | | 2 (5.00) | |  |

| **Characteristic** | | **Palencia**  **n= 40**  **Cases (%)** | **San José Del Golfo**  **n= 45**  **Cases (%)** | **San José Pinula**  **n= 39**  **Cases (%)** | **San Juan Sacatepéquez**  **n= 40**  **Cases (%)** | **San Miguel Petapa**  **n= 45**  **Cases (%)** | **San Pedro Ayampuc**  **n= 45**  **Cases (%)** | **San Pedro Sacatepéquez**  **n= 45**  **Cases (%)** |
| --- | --- | --- | --- | --- | --- | --- | --- | --- |
| Language | Unknown | 0 (0.00) | 2 (4.44) | 0 (0.00) | 0 (0.00) | 0 (0.00) | 5 (11.11) | 0 (0.00) |
|  | Spanish | 40 (100.00) | 43 (95.56) | 39 (100.00) | 27 (67.50) | 41 (91.11) | 40 (88.89) | 39 (86.67) |
|  | Mayan | 0 (0.00) | 0 (0.00) | 0 (0.00) | 13 (32.50) | 4 (8.89) | 0 (0.00) | 6 (13.33) |
| Maize Consumption | ≤4750 g/week | 36 (90.00) | 45 (100.00) | 37 (94.87) | 27 (67.50) | 42 (93.33) | 42 (93.33) | 36 (80.00) |
|  | >4750 g/week | 4 (10.00) | 0 (0.00) | 2 (5.13) | 13 (32.50) | 3 (6.67) | 3 (6.67) | 9 (20.00) |
| uFB_1_ level | Low (<0.5 ng/ml) | 29 (72.50) | 32 (71.11) | 29 (74.36) | 24 (60.00) | 34 (75.56) | 29 (64.44) | 25 (55.56) |
|  | High (≥0.5 ng/ml) | 11 (27.50) | 13 (28.89) | 10 (25.64) | 16 (40.00) | 11 (24.44) | 16 (35.56) | 20 (44.44) |
| Age group | Less than 25 years | 28 (70.00) | 16 (35.56) | 17 (43.59) | 19 (47.50) | 21 (46.67) | 14 (31.11) | 22 (48.89) |
|  | 25 to 29 years | 2 (5.00) | 5 (11.11) | 8 (20.51) | 9 (22.50) | 12 (26.67) | 8 (17.78) | 6 (13.33) |
|  | 30 to 34 years | 1 (2.50) | 12 (26.67) | 8 (20.51) | 8 (20.00) | 8 (17.78) | 5 (11.11) | 12 (26.67) |
|  | 35 years and above | 9 (22.50) | 12 (26.67) | 6 (15.38) | 4 (10.00) | 4 (8.89) | 18 (40.00) | 5 (11.11) |
| Education | Unknown | 0 (0.00) | 1 (2.22) | 0 (0.00) | 1 (2.50) | 0 (0.00) | 0 (0.00) | 0 (0.00) |
|  | Less than high school | 23 (57.50) | 21 (46.67) | 23 (58.97) | 31 (77.50) | 24 (53.33) | 25 (55.56) | 26 (57.78) |
|  | High school and above | 17 (42.50) | 23 (51.11) | 16 (41.03) | 8 (20.00) | 21 (46.67) | 20 (44.44) | 19 (42.22) |
| Occupation | Unknown | 0 (0.00) | 0 (0.00) | 0 (0.00) | 0 (0.00) | 0 (0.00) | 0 (0.00) | 0 (0.00) |
|  | Housewife | 38 (95.00) | 40 (88.89) | 38 (97.44) | 31 (77.50) | 38 (84.44) | 35 (77.78) | 36 (80.00) |
|  | Paid worker | 2 (5.00) | 5 (11.11) | 1 (2.56) | 9 (22.50) | 7 (15.56) | 10 (22.22) | 9 (20.00) |
| Number persons in household | Unknown | 0 (0.00) | 0 (0.00) | 0 (0.00) | 0 (0.00) | 0 (0.00) | 1 (2.22) | 0 (0.00) |
|  | 1 to 7 persons | 38 (95.00) | 42 (93.33) | 37 (94.87) | 35 (87.50) | 45 (100.00) | 39 (86.67) | 44 (97.78) |
|  | 8 to 18 persons | 2 (5.00) | 3 (6.67) | 2 (5.13) | 5 (12.50) | 0 (0.00) | 5 (11.11) | 1 (2.22) |

| **Characteristic** | | **San Raymundo**  **n= 40**  **Cases (%)** | **Santa Catarina Pinula**  **n= 40**  **Cases (%)** | **Villa Canales**  **n= 45**  **Cases (%)** | **Villa Nueva**  **n= 45**  **Cases (%)** | **P-value*** |
| --- | --- | --- | --- | --- | --- | --- |
| Language | Unknown | 0 (0.00) | 0 (0.00) | 0 (0.00) | 0 (0.00) | <0.001 |
|  | Spanish | 37 (92.50) | 43 (97.73) | 41 (91.11) | 43 (95.56) | <0.001 |
|  | Mayan | 3 (7.50) | 1 (2.27) | 4 (8.89) | 2 (4.44) | <0.001 |
| Maize Consumption | ≤4750 g/week | 36 (90.00) | 40 (90.91) | 38 (84.44) | 41 (91.11) | <0.001 |
|  | >4750 g/week | 4 (10.00) | 4 (9.09) | 7 (15.56) | 4 (8.89) | <0.001 |
| uFB_1_ level | Low (<0.5 ng/ml) | 31 (77.50) | 36 (81.82) | 33 (73.33) | 33 (73.33) | <0.001 |
|  | High (≥0.5 ng/ml) | 9 (22.50) | 8 (18.18) | 12 (26.67) | 12 (26.67) | <0.001 |
| Age group | Less than 25 years | 21 (52.50) | 21 (47.73) | 20 (44.44) | 30 (66.67) | <0.001 |
|  | 25 to 29 years | 11 (27.50) | 8 (18.18) | 10 (22.22) | 8 (17.78) | <0.001 |
|  | 30 to 34 years | 4 (10.00) | 7 (15.91) | 12 (26.67) | 4 (8.89) | <0.001 |
|  | 35 years and above | 4 (10.00) | 8 (18.18) | 3 (6.67) | 3 (6.67) | <0.001 |
| Education | Unknown | 0 (0.00) | 0 (0.00) | 0 (0.00) | 0 (0.00) | <0.001 |
|  | Less than high school | 19 (47.50) | 14 (31.82) | 20 (44.44) | 20 (44.44) | <0.001 |
|  | High school and above | 21 (52.50) | 30 (68.18) | 25 (55.56) | 25 (55.56) | <0.001 |
| Occupation | Unknown | 0 (0.00) | 0 (0.00) | 0 (0.00) | 0 (0.00) | <0.001 |
|  | Housewife | 32 (80.00) | 34 (77.27) | 30 (66.67) | 34 (75.56) | <0.001 |
|  | Paid worker | 8 (20.00) | 10 (22.73) | 15 (33.33) | 11 (24.44) | <0.001 |
| Number persons in household | Unknown | 0 (0.00) | 0 (0.00) | 0 (0.00) | 0 (0.00) | <0.001 |
|  | 1 to 7 persons | 30 (75.00) | 34 (77.27) | 42 (93.33) | 45 (100.00) | <0.001 |
|  | 8 to 18 persons | 10 (25.00) | 10 (22.73) | 3 (6.67) | 0 (0.00) | <0.001 |

*p-value calculated using Kruskal-Wallis test
