# Supplementary material for: Dietary and socioeconomic risk factors for fumonisin exposure among women of reproductive age in 18 municipalities in Guatemala from 2013 to 2014
Source: PLOS Glob Public Health. 2022 Aug 9;2(8):e0000337. doi: 10.1371/journal.pgph.0000337 (PMC10021672; doi:10.1371/journal.pgph.0000337)
Supplement: S3 Table — (DOCX) [file pgph.0000337.s004.docx]

**S3 Table** Mean and median intakes (servings) of food items by FB_1_ exposure group.

|  | **Overall** | | | | **Low (<0.5 ng/ml)** | | | | **High (=>0.5 ng/ml)** | | | | **P-value** |
| --- | --- | --- | --- | --- | --- | --- | --- | --- | --- | --- | --- | --- | --- |
|  | **N** | **Mean** | **± Std** | **Median** | **N** | **Mean** | **± Std** | **Median** | **N** | **Mean** | **± Std** | **Median** |  |
| Tortillas | 773 | 58.70 | ± 36.22 | 56.00 | 530 | 53.77 | ± 34.71 | 49.00 | 243 | 69.46 | ± 37.17 | 63.00 | <0.001 |
| French bread | 775 | 16.45 | ± 18.84 | 12.00 | 532 | 17.55 | ± 17.98 | 14.00 | 243 | 14.05 | ± 20.44 | 8.00 | <0.001 |
| Corn flakes | 773 | 1.25 | ± 2.28 | 0.00 | 530 | 1.41 | ± 2.43 | 0.00 | 243 | 0.92 | ± 1.87 | 0.00 | 0.003 |
| Macuy | 772 | 0.47 | ± 0.85 | 0.00 | 531 | 0.43 | ± 0.90 | 0.00 | 241 | 0.54 | ± 0.75 | 0.00 | 0.003 |
| Tayuyos | 775 | 0.22 | ± 1.08 | 0.00 | 532 | 0.13 | ± 0.85 | 0.00 | 243 | 0.42 | ± 1.44 | 0.00 | 0.003 |
| Lettuce | 775 | 0.54 | ± 1.56 | 0.00 | 532 | 0.63 | ± 1.76 | 0.00 | 243 | 0.36 | ± 0.97 | 0.00 | 0.006 |
| Pinol | 775 | 0.21 | ± 0.98 | 0.00 | 532 | 0.19 | ± 1.04 | 0.00 | 243 | 0.25 | ± 0.82 | 0.00 | 0.024 |
| Green peas | 775 | 0.12 | ± 0.75 | 0.00 | 532 | 0.14 | ± 0.87 | 0.00 | 243 | 0.06 | ± 0.36 | 0.00 | 0.031 |
| Beef | 775 | 3.58 | ± 4.49 | 3.00 | 532 | 3.77 | ± 4.51 | 4.00 | 243 | 3.16 | ± 4.42 | 2.00 | 0.033 |
| Onion | 774 | 1.26 | ± 2.30 | 1.00 | 531 | 1.16 | ± 1.53 | 1.00 | 243 | 1.49 | ± 3.43 | 1.00 | 0.050 |
| Whole milk | 774 | 1.42 | ± 3.32 | 0.00 | 531 | 1.56 | ± 3.66 | 0.00 | 243 | 1.12 | ± 2.38 | 0.00 | 0.052 |
| Papaya | 775 | 0.18 | ± 0.47 | 0.00 | 532 | 0.20 | ± 0.52 | 0.00 | 243 | 0.14 | ± 0.35 | 0.00 | 0.054 |
| Beans | 775 | 22.09 | ± 44.20 | 16.00 | 532 | 22.66 | ± 50.00 | 11.97 | 243 | 20.85 | ± 27.60 | 16.00 | 0.064 |
| Peanuts | 772 | 1.55 | ± 28.86 | 0.00 | 529 | 0.58 | ± 2.43 | 0.00 | 243 | 3.64 | ± 51.33 | 0.00 | 0.089 |
| Green beans | 775 | 0.37 | ± 0.89 | 0.00 | 532 | 0.36 | ± 0.96 | 0.00 | 243 | 0.39 | ± 0.73 | 0.00 | 0.090 |
| Tangerine | 774 | 0.70 | ± 2.69 | 0.00 | 531 | 0.64 | ± 2.64 | 0.00 | 243 | 0.83 | ± 2.79 | 0.00 | 0.120 |
| Chard | 774 | 0.14 | ± 0.43 | 0.00 | 532 | 0.12 | ± 0.38 | 0.00 | 242 | 0.18 | ± 0.52 | 0.00 | 0.121 |
| Chicken | 775 | 8.02 | ± 7.93 | 8.00 | 532 | 8.06 | ± 7.54 | 8.00 | 243 | 7.93 | ± 8.76 | 4.00 | 0.134 |
| Haba grano seco | 775 | 0.11 | ± 0.79 | 0.00 | 532 | 0.11 | ± 0.55 | 0.00 | 243 | 0.12 | ± 1.15 | 0.00 | 0.135 |
| Pork | 775 | 2.05 | ± 28.83 | 0.00 | 532 | 2.58 | ± 34.72 | 0.00 | 243 | 0.89 | ± 3.51 | 0.00 | 0.137 |
| Powdered milk | 775 | 0.80 | ± 3.21 | 0.00 | 532 | 0.96 | ± 3.73 | 0.00 | 243 | 0.45 | ± 1.54 | 0.00 | 0.139 |
| Boiled sweet corn | 775 | 0.71 | ± 2.25 | 0.00 | 532 | 0.77 | ± 2.54 | 0.00 | 243 | 0.58 | ± 1.44 | 0.00 | 0.160 |
| Maize coffee | 775 | 1.69 | ± 5.55 | 0.00 | 532 | 1.32 | ± 4.17 | 0.00 | 243 | 2.49 | ± 7.70 | 0.00 | 0.163 |
| Masa beverage | 775 | 0.94 | ± 3.30 | 0.00 | 532 | 0.94 | ± 3.40 | 0.00 | 243 | 0.93 | ± 3.08 | 0.00 | 0.204 |
| Beef kidney | 775 | 0.11 | ± 1.17 | 0.00 | 532 | 0.13 | ± 1.23 | 0.00 | 243 | 0.08 | ± 1.06 | 0.00 | 0.214 |
| Starch atol | 775 | 0.27 | ± 1.44 | 0.00 | 532 | 0.33 | ± 1.67 | 0.00 | 243 | 0.14 | ± 0.68 | 0.00 | 0.260 |
| Tortrix | 774 | 0.78 | ± 1.90 | 0.00 | 531 | 0.84 | ± 2.11 | 0.00 | 243 | 0.65 | ± 1.33 | 0.00 | 0.278 |
| Chicken liver | 775 | 0.40 | ± 1.38 | 0.00 | 532 | 0.37 | ± 1.29 | 0.00 | 243 | 0.45 | ± 1.56 | 0.00 | 0.351 |
| Beef liver | 775 | 0.41 | ± 1.87 | 0.00 | 532 | 0.41 | ± 1.89 | 0.00 | 243 | 0.40 | ± 1.83 | 0.00 | 0.389 |
| Banana | 773 | 3.78 | ± 5.71 | 2.00 | 531 | 3.89 | ± 6.02 | 2.00 | 242 | 3.54 | ± 4.95 | 2.00 | 0.408 |
| Tomato | 775 | 2.04 | ± 2.46 | 2.00 | 532 | 2.12 | ± 2.54 | 2.00 | 243 | 1.87 | ± 2.28 | 2.00 | 0.418 |
| Tostadas | 775 | 1.09 | ± 3.02 | 0.00 | 532 | 0.97 | ± 1.80 | 0.00 | 243 | 1.36 | ± 4.69 | 0.00 | 0.421 |
| Oil | 773 | 6.60 | ± 18.16 | 3.75 | 532 | 7.32 | ± 21.48 | 3.75 | 241 | 5.03 | ± 6.00 | 3.75 | 0.428 |
| Soy flour | 775 | 0.04 | ± 0.36 | 0.00 | 532 | 0.04 | ± 0.41 | 0.00 | 243 | 0.02 | ± 0.19 | 0.00 | 0.434 |
| Spinach | 774 | 0.07 | ± 0.37 | 0.00 | 532 | 0.08 | ± 0.42 | 0.00 | 242 | 0.05 | ± 0.23 | 0.00 | 0.464 |
| Chuchitos | 775 | 0.84 | ± 3.24 | 0.00 | 532 | 0.92 | ± 3.79 | 0.00 | 243 | 0.66 | ± 1.42 | 0.00 | 0.481 |
| Bledo | 772 | 0.12 | ± 0.42 | 0.00 | 529 | 0.12 | ± 0.44 | 0.00 | 243 | 0.13 | ± 0.37 | 0.00 | 0.488 |
| Orange | 775 | 1.48 | ± 3.47 | 0.00 | 532 | 1.57 | ± 3.60 | 0.00 | 243 | 1.28 | ± 3.16 | 0.00 | 0.518 |
| Water cress | 775 | 0.20 | ± 0.51 | 0.00 | 532 | 0.21 | ± 0.52 | 0.00 | 243 | 0.19 | ± 0.48 | 0.00 | 0.532 |
| Beet | 775 | 0.24 | ± 0.78 | 0.00 | 532 | 0.25 | ± 0.78 | 0.00 | 243 | 0.21 | ± 0.77 | 0.00 | 0.534 |
| Avocado | 775 | 0.86 | ± 1.42 | 0.50 | 532 | 0.83 | ± 1.42 | 0.50 | 243 | 0.91 | ± 1.42 | 0.50 | 0.540 |
| Sugar | 775 | 45.83 | ± 122.30 | 32.86 | 532 | 41.27 | ± 37.92 | 32.86 | 243 | 55.80 | ± 211.04 | 32.86 | 0.559 |
| Cream | 773 | 2.00 | ± 3.88 | 0.00 | 530 | 2.06 | ± 3.82 | 0.00 | 243 | 1.87 | ± 4.01 | 0.00 | 0.560 |
| Polenta (corn flour) | 775 | 0.18 | ± 1.64 | 0.00 | 532 | 0.17 | ± 1.65 | 0.00 | 243 | 0.20 | ± 1.63 | 0.00 | 0.594 |
| Cheese | 773 | 1.41 | ± 2.67 | 0.00 | 531 | 1.44 | ± 2.56 | 0.00 | 242 | 1.35 | ± 2.89 | 0.00 | 0.602 |
| Broccoli | 775 | 0.46 | ± 1.06 | 0.00 | 532 | 0.48 | ± 1.08 | 0.00 | 243 | 0.43 | ± 1.03 | 0.00 | 0.603 |
| Eggs | 775 | 4.16 | ± 4.14 | 3.00 | 532 | 4.20 | ± 4.36 | 3.00 | 243 | 4.09 | ± 3.62 | 3.00 | 0.637 |
| Tamales | 774 | 0.61 | ± 1.12 | 0.00 | 531 | 0.59 | ± 1.00 | 0.00 | 243 | 0.64 | ± 1.34 | 0.00 | 0.656 |
| Cauliflower | 775 | 0.18 | ± 0.61 | 0.00 | 532 | 0.17 | ± 0.56 | 0.00 | 243 | 0.22 | ± 0.70 | 0.00 | 0.675 |
| Cabbage | 775 | 0.48 | ± 4.19 | 0.00 | 532 | 0.37 | ± 3.42 | 0.00 | 243 | 0.73 | ± 5.52 | 0.00 | 0.707 |
| Nachos | 772 | 0.35 | ± 1.36 | 0.00 | 529 | 0.35 | ± 1.41 | 0.00 | 243 | 0.36 | ± 1.25 | 0.00 | 0.716 |
| Tacos | 775 | 0.44 | ± 1.96 | 0.00 | 532 | 0.37 | ± 1.20 | 0.00 | 243 | 0.58 | ± 3.01 | 0.00 | 0.767 |
| Haba flour | 775 | 0.19 | ± 1.26 | 0.00 | 532 | 0.16 | ± 0.92 | 0.00 | 243 | 0.26 | ± 1.80 | 0.00 | 0.776 |
| Sweet bread | 773 | 8.03 | ± 10.81 | 4.00 | 530 | 8.13 | ± 10.98 | 4.00 | 243 | 7.79 | ± 10.45 | 4.00 | 0.781 |
| Asparagus | 775 | 0.28 | ± 1.71 | 0.00 | 532 | 0.26 | ± 1.42 | 0.00 | 243 | 0.34 | ± 2.20 | 0.00 | 0.858 |
| Incaparina | 775 | 2.53 | ± 5.06 | 0.00 | 532 | 2.54 | ± 5.12 | 0.00 | 243 | 2.51 | ± 4.95 | 0.00 | 0.869 |
| Rice | 775 | 10.86 | ± 14.35 | 8.00 | 532 | 10.88 | ± 15.00 | 8.00 | 243 | 10.80 | ± 12.85 | 8.00 | 0.888 |
| Fish | 775 | 1.18 | ± 3.61 | 0.00 | 532 | 1.25 | ± 3.84 | 0.00 | 243 | 1.02 | ± 3.05 | 0.00 | 0.910 |
| Tamalitos | 775 | 0.69 | ± 2.04 | 0.00 | 532 | 0.66 | ± 1.97 | 0.00 | 243 | 0.75 | ± 2.19 | 0.00 | 0.911 |
| Lard | 775 | 0.28 | ± 1.32 | 0.00 | 532 | 0.33 | ± 1.49 | 0.00 | 243 | 0.19 | ± 0.85 | 0.00 | 0.948 |
| Corn atole (sweet beverage) | 773 | 0.33 | ± 1.39 | 0.00 | 530 | 0.35 | ± 1.57 | 0.00 | 243 | 0.29 | ± 0.90 | 0.00 | 0.985 |

*p-value calculated using chi-square test
