# Supplementary material for: Dietary and socioeconomic risk factors for fumonisin exposure among women of reproductive age in 18 municipalities in Guatemala from 2013 to 2014
Source: PLOS Glob Public Health. 2022 Aug 9;2(8):e0000337. doi: 10.1371/journal.pgph.0000337 (PMC10021672; doi:10.1371/journal.pgph.0000337)
Supplement: S4 Table — (DOCX) [file pgph.0000337.s005.docx]

**S4 Table** Consumption of food groups by department.

|  |  | **Overall** | | | **Alta Verapaz (Cobán)** | | | **Guatemala** | | | **P-value*** |
| --- | --- | --- | --- | --- | --- | --- | --- | --- | --- | --- | --- |
|  |  | **N** | **Mean ± Std** | **Median** | **N** | **Mean ± Std** | **Median** | **N** | **Mean ± Std** | **Median** |  |
| Summary Statistics | Maize-based food consumption (g) | 766 | 3021.10 ± 1654.34 | 2793.50 | 45 | 4506.22 ± 1368.03 | 4660.00 | 721 | 2928.41 ± 1626.98 | 2700.00 | <0.001 |
|  | Total grams consumed | 749 | 7412.05 ± 4037.97 | 6654.50 | 45 | 12337.88 ± 10409.75 | 8416.50 | 704 | 7097.19 ± 2985.65 | 6543.13 | <0.001 |
|  | Percent of total food maize-based | 749 | 41.85 ± 16.00 | 42.04 | 45 | 49.20 ± 20.31 | 50.37 | 704 | 41.38 ± 15.58 | 41.72 | 0.004 |
|  | Number of food types consumed | 775 | 20.99 ± 5.93 | 20.00 | 45 | 19.18 ± 4.97 | 18.00 | 730 | 21.10 ± 5.97 | 21.00 | 0.022 |
|  | Number of maize-based foods | 775 | 4.87 ± 2.31 | 4.00 | 45 | 5.18 ± 2.18 | 4.00 | 730 | 4.85 ± 2.31 | 4.00 | 0.369 |
| Food group (servings) | Dairy | 770 | 5.63 ± 7.26 | 3.3 | 45 | 2.3 ± 2.98 | 1.39 | 725 | 5.84 ± 7.40 | 4 | <0.001 |
|  | Bread | 773 | 24.46 ± 23.13 | 20 | 45 | 5.24 ± 9.79 | 2 | 728 | 25.65 ± 23.20 | 21 | <0.001 |
|  | Highly processed maize-based foods | 773 | 2.3 ± 3.62 | 1 | 45 | 1.06 ± 2.11 | 0 | 728 | 2.38 ± 3.68 | 1 | <0.001 |
|  | Sugar | 775 | 45.83 ± 122.30 | 32.86 | 45 | 96.75 ± 486.80 | 21 | 730 | 42.69 ± 37.52 | 32.86 | <0.001 |
|  | Fats and oils | 773 | 6.89 ± 18.27 | 3.75 | 45 | 2.56 ± 4.72 | 1 | 728 | 7.16 ± 18.75 | 4 | <0.001 |
|  | Other vegetables | 774 | 4.2 ± 5.62 | 3 | 45 | 11.89 ± 16.97 | 5 | 729 | 3.73 ± 3.50 | 3 | <0.001 |
|  | Green and yellow vegetables | 775 | 2.09 ± 2.89 | 1 | 45 | 0.9 ± 1.45 | 0 | 730 | 2.17 ± 2.94 | 1.15 | <0.001 |
|  | Chicken | 775 | 8.42 ± 8.06 | 8 | 45 | 3.76 ± 3.77 | 3 | 730 | 8.7 ± 8.16 | 8 | <0.001 |
|  | Locally produced maize-based foods | 767 | 67.14 ± 39.62 | 63 | 45 | 113.28 ± 32.86 | 117.95 | 722 | 64.26 ± 38.22 | 60 | <0.001 |
|  | Beef food group | 775 | 4.1 ± 5.11 | 4 | 45 | 1.8 ± 1.93 | 1 | 730 | 4.24 ± 5.21 | 4 | 0.002 |
|  | Eggs | 775 | 4.16 ± 4.14 | 3 | 45 | 3.22 ± 4.73 | 2 | 730 | 4.22 ± 4.10 | 3 | 0.006 |
|  | Rice | 775 | 10.86 ± 14.35 | 8 | 45 | 21.11 ± 21.23 | 16 | 730 | 10.23 ± 13.59 | 8 | 0.013 |
|  | Fruit | 772 | 6.15 ± 8.62 | 3.5 | 45 | 8.06 ± 9.10 | 6 | 727 | 6.03 ± 8.59 | 3.25 | 0.080 |
|  | Green leafy vegetables | 770 | 1.54 ± 2.28 | 1 | 45 | 1.46 ± 1.11 | 1.15 | 725 | 1.55 ± 2.34 | 1 | 0.118 |
|  | Grains | 775 | 22.43 ± 44.29 | 16 | 45 | 43.74 ± 120.62 | 17.95 | 730 | 21.12 ± 34.27 | 16 | 0.128 |
|  | Nuts | 772 | 1.55 ± 28.86 | 0 | 45 | 18.29 ± 119.20 | 0 | 727 | 0.51 ± 2.21 | 0 | 0.208 |
|  | Micronutrient fortified, maize-based food | 775 | 2.53 ± 5.06 | 0 | 45 | 1.51 ± 2.78 | 0 | 730 | 2.59 ± 5.16 | 0 | 0.495 |
|  | Pork | 775 | 2.05 ± 28.83 | 0 | 45 | 18.42 ± 119.17 | 0 | 730 | 1.04 ± 2.81 | 0 | 0.716 |
|  | Fish | 775 | 1.18 ± 3.61 | 0 | 45 | 0.8 ± 2.64 | 0 | 730 | 1.2 ± 3.66 | 0 | 0.807 |

*p-value calculated using Kruskal-Wallis test
