# Supplementary material for: Dietary and socioeconomic risk factors for fumonisin exposure among women of reproductive age in 18 municipalities in Guatemala from 2013 to 2014
Source: PLOS Glob Public Health. 2022 Aug 9;2(8):e0000337. doi: 10.1371/journal.pgph.0000337 (PMC10021672; doi:10.1371/journal.pgph.0000337)
Supplement: S5 Table — (DOCX) [file pgph.0000337.s006.docx]

**S5 Table** Mean and median consumption of food groups by municipality.

|  | | **Overall** | | | **Amatitlán** | | | **Chinautla** | | |
| --- | --- | --- | --- | --- | --- | --- | --- | --- | --- | --- |
|  |  | **N** | **Mean ± Std** | **Median** | **N** | **Mean ± Std** | **Median** | **N** | **Mean ± Std** | **Median** |
| Summary statistics | Maize-based food consumption (g) | 766 | 3,021.10 ± 1,654.34 | 2793.50 | 42 | 2,116.38 ± 1,468.01 | 1695.00 | 44 | 2,870.97 ± 1,462.60 | 2691.00 |
|  | Percent of total food maize-based | 749 | 41.85 ± 16.00 | 42.04 | 41 | 37.25 ± 15.90 | 35.53 | 40 | 37.25 ± 11.38 | 37.30 |
|  | Total grams consumed | 749 | 7,412.05 ± 4,037.97 | 6654.50 | 41 | 5,562.35 ± 2,263.50 | 5267.10 | 40 | 7,794.58 ± 3,377.28 | 7313.63 |
|  | Number of food types consumed | 775 | 20.99 ± 5.93 | 20.00 | 42 | 19.90 ± 5.58 | 19.50 | 45 | 21.53 ± 5.21 | 21.00 |
|  | Number of maize-based foods | 775 | 4.87 ± 2.31 | 4.00 | 42 | 4.43 ± 2.19 | 4.00 | 45 | 4.91 ± 2.58 | 5.00 |
| Food group (servings) | Eggs | 775 | 8.42 ± 8.06 | 8.00 | 42 | 7.95 ± 5.61 | 8.00 | 45 | 9.31 ± 10.21 | 8.00 |
|  | Dairy | 775 | 2.57 ± 28.90 | 0.00 | 42 | 0.90 ± 2.02 | 0.00 | 45 | 1.93 ± 5.22 | 0.00 |
|  | Bread | 770 | 9.79 ± 8.60 | 7.47 | 42 | 8.69 ± 9.06 | 6.00 | 44 | 13.50 ± 13.06 | 10.59 |
|  | Rice | 773 | 1.25 ± 2.28 | 0.00 | 42 | 0.97 ± 1.73 | 0.00 | 45 | 1.87 ± 3.03 | 0.47 |
|  | Grains | 773 | 24.46 ± 23.13 | 20.00 | 42 | 24.71 ± 20.16 | 21.00 | 45 | 34.18 ± 24.70 | 31.00 |
|  | Nuts | 775 | 10.86 ± 14.35 | 8.00 | 42 | 7.42 ± 8.26 | 4.99 | 45 | 10.73 ± 15.24 | 4.00 |
|  | Micronutrient fortified, maize-based food | 775 | 22.43 ± 44.29 | 16.00 | 42 | 15.87 ± 15.73 | 12.19 | 45 | 22.45 ± 31.04 | 11.97 |
|  | Highly processed maize-based foods | 774 | 2.36 ± 4.45 | 1.00 | 42 | 0.98 ± 1.83 | 0.00 | 45 | 1.74 ± 2.30 | 1.00 |
|  | Fats and oils | 772 | 1.13 ± 2.60 | 0.00 | 42 | 1.26 ± 2.08 | 0.00 | 44 | 1.16 ± 2.27 | 0.00 |
|  | Fruit | 773 | 0.60 ± 2.02 | 0.00 | 42 | 0.55 ± 1.33 | 0.00 | 45 | 1.58 ± 4.44 | 0.00 |
|  | Other vegetables | 775 | 45.83 ± 122.30 | 32.86 | 42 | 40.43 ± 28.95 | 32.86 | 45 | 45.94 ± 38.98 | 32.86 |
|  | Green and yellow vegetables | 770 | 8.46 ± 34.14 | 4.00 | 42 | 5.47 ± 6.60 | 3.00 | 44 | 10.04 ± 18.20 | 5.00 |
|  | Green leafy vegetables | 772 | 6.15 ± 8.62 | 3.50 | 41 | 4.89 ± 5.33 | 4.00 | 45 | 7.33 ± 8.15 | 5.00 |
|  | Fish | 774 | 4.20 ± 5.62 | 3.00 | 42 | 2.99 ± 2.58 | 3.00 | 45 | 5.09 ± 4.53 | 4.00 |
|  | Chicken | 775 | 2.09 ± 2.89 | 1.00 | 42 | 1.42 ± 1.44 | 1.00 | 45 | 3.19 ± 5.35 | 2.00 |
|  | Pork | 770 | 1.54 ± 2.28 | 1.00 | 42 | 0.76 ± 1.01 | 0.04 | 42 | 1.53 ± 1.73 | 1.08 |
|  | Beef food group | 775 | 1.18 ± 3.61 | 0.00 | 42 | 2.39 ± 4.72 | 0.00 | 45 | 0.92 ± 2.69 | 0.00 |
|  | Locally produced maize-based foods | 772 | 67.60 ± 39.96 | 63.00 | 42 | 46.49 ± 35.99 | 34.69 | 45 | 60.77 ± 33.08 | 58.00 |

|  | | **Chuarrancho** | | | | **Cobán** | | | **Fraijanes** | |
| --- | --- | --- | --- | --- | --- | --- | --- | --- | --- | --- |
|  |  | **N** | **Mean ± Std** | **Median** | **N** | **Mean ± Std** | **Median** | **N** | **Mean ± Std** | **Median** |
| Summary statistics | Maize-based food consumption (g) | 45 | 3,744.44 ± 1,936.63 | 3,565.00 | 45 | 4,506.22 ± 1,368.03 | 4660.00 | 45 | 3,534.08 ± 1,930.81 | 3,388.50 |
|  | Percent of total food maize-based | 45 | 49.70 ± 15.02 | 51.37 | 45 | 49.20 ± 20.31 | 50.37 | 45 | 49.10 ± 15.43 | 52.05 |
|  | Total grams consumed | 45 | 7,419.69 ± 2,654.09 | 6,831.25 | 45 | 12,337.88 ± 10,409.75 | 8,416.50 | 45 | 7,161.81 ± 3,204.39 | 6,201.75 |
|  | Number of food types consumed | 45 | 22.42 ± 6.98 | 22.00 | 45 | 19.18 ± 4.97 | 18.00 | 45 | 19.73 ± 6.78 | 19.00 |
|  | Number of maize-based foods | 45 | 5.44 ± 2.89 | 4.00 | 45 | 5.18 ± 2.18 | 4.00 | 45 | 4.96 ± 2.57 | 4.00 |
| Food group (servings) | Eggs | 45 | 8.00 ± 7.70 | 4.00 | 45 | 3.76 ± 3.77 | 3.00 | 45 | 7.09 ± 6.28 | 8.00 |
|  | Dairy | 45 | 1.42 ± 2.35 | 0.00 | 45 | 18.42 ± 119.17 | 0.00 | 45 | 1.31 ± 3.60 | 0.00 |
|  | Bread | 45 | 8.14 ± 7.61 | 6.00 | 45 | 5.52 ± 5.92 | 4.00 | 45 | 7.50 ± 6.39 | 4.57 |
|  | Rice | 45 | 0.71 ± 1.59 | 0.00 | 45 | 0.20 ± 0.92 | 0.00 | 45 | 1.71 ± 2.39 | 0.00 |
|  | Grains | 45 | 20.84 ± 20.93 | 14.00 | 45 | 5.24 ± 9.79 | 2.00 | 45 | 16.31 ± 16.10 | 12.00 |
|  | Nuts | 45 | 7.63 ± 8.13 | 8.00 | 45 | 21.11 ± 21.23 | 16.00 | 45 | 8.35 ± 8.72 | 5.98 |
|  | Micronutrient fortified, maize-based food | 45 | 17.84 ± 30.03 | 9.25 | 45 | 43.74 ± 120.62 | 17.95 | 45 | 14.09 ± 14.70 | 8.97 |
|  | Highly processed maize-based foods | 45 | 5.36 ± 12.85 | 2.00 | 45 | 3.98 ± 4.22 | 3.00 | 45 | 2.98 ± 4.73 | 1.00 |
|  | Fats and oils | 45 | 0.76 ± 1.35 | 0.00 | 45 | 1.18 ± 3.12 | 0.00 | 45 | 1.56 ± 3.63 | 0.00 |
|  | Fruit | 45 | 1.20 ± 2.00 | 0.00 | 45 | 0.56 ± 1.67 | 0.00 | 45 | 0.20 ± 0.76 | 0.00 |
|  | Other vegetables | 45 | 45.59 ± 37.39 | 32.86 | 45 | 96.75 ± 486.80 | 21.00 | 45 | 38.70 ± 26.27 | 32.86 |
|  | Green and yellow vegetables | 45 | 4.40 ± 4.32 | 3.00 | 45 | 20.85 ± 119.67 | 2.00 | 45 | 6.00 ± 9.81 | 3.00 |
|  | Green leafy vegetables | 45 | 5.61 ± 8.15 | 3.00 | 45 | 8.06 ± 9.10 | 6.00 | 45 | 7.83 ± 10.31 | 4.00 |
|  | Fish | 45 | 3.75 ± 2.75 | 3.00 | 45 | 11.89 ± 16.97 | 5.00 | 45 | 3.77 ± 2.57 | 3.00 |
|  | Chicken | 45 | 2.43 ± 2.70 | 1.38 | 45 | 0.90 ± 1.45 | 0.00 | 45 | 3.13 ± 4.16 | 2.00 |
|  | Pork | 45 | 1.92 ± 2.22 | 1.15 | 45 | 1.46 ± 1.11 | 1.15 | 45 | 1.80 ± 3.94 | 0.72 |
|  | Beef food group | 45 | 0.72 ± 1.82 | 0.00 | 45 | 0.80 ± 2.64 | 0.00 | 45 | 0.56 ± 2.39 | 0.00 |
|  | Locally produced maize-based foods | 45 | 84.62 ± 41.74 | 80.00 | 45 | 112.60 ± 33.26 | 118.00 | 45 | 78.55 ± 46.97 | 71.50 |

|  |  | **Guatemala** | | | **Mixco** | | | **Palencia** | | |
| --- | --- | --- | --- | --- | --- | --- | --- | --- | --- | --- |
|  |  | **N** | **Mean ± Std** | **Median** | **N** | **Mean ± Std** | **Median** | **N** | **Mean ± Std** | **Median** |
| Summary statistics | Maize-based food consumption (g) | 40 | 2,427.29 ± 1,520.78 | 1,982.50 | 39 | 2,587.23 ± 1,560.71 | 2,375.00 | 39 | 2,815.77 ± 1,775.42 | 2,520.00 |
|  | Percent of total food maize-based | 38 | 39.73 ± 17.92 | 36.66 | 37 | 38.06 ± 16.28 | 37.07 | 39 | 41.55 ± 15.30 | 43.76 |
|  | Total grams consumed | 38 | 5,959.05 ± 1,943.22 | 5,616.63 | 37 | 6,601.30 ± 2,286.77 | 6,393.50 | 39 | 7,145.42 ± 4,390.69 | 5,858.75 |
|  | Number of food types consumed | 40 | 19.78 ± 5.28 | 19.00 | 40 | 21.88 ± 4.10 | 22.00 | 40 | 19.63 ± 6.69 | 19.00 |
|  | Number of maize-based foods | 40 | 4.25 ± 1.63 | 4.00 | 40 | 5.03 ± 1.87 | 5.00 | 40 | 4.63 ± 2.37 | 4.00 |
| Food group (servings) | Eggs | 40 | 9.65 ± 7.66 | 8.00 | 40 | 7.63 ± 5.95 | 8.00 | 40 | 11.20 ± 7.95 | 8.50 |
|  | Dairy | 40 | 1.50 ± 2.61 | 0.00 | 40 | 1.88 ± 2.64 | 0.00 | 40 | 2.28 ± 5.51 | 0.00 |
|  | Bread | 39 | 10.57 ± 7.34 | 9.00 | 39 | 10.58 ± 9.39 | 7.50 | 40 | 9.64 ± 8.26 | 7.31 |
|  | Rice | 40 | 0.63 ± 1.26 | 0.00 | 40 | 1.48 ± 2.15 | 0.00 | 40 | 0.68 ± 1.58 | 0.00 |
|  | Grains | 40 | 31.95 ± 22.87 | 27.00 | 39 | 26.67 ± 23.07 | 22.00 | 40 | 20.33 ± 17.88 | 14.00 |
|  | Nuts | 40 | 11.24 ± 13.60 | 8.00 | 40 | 11.57 ± 15.62 | 8.00 | 40 | 8.40 ± 11.64 | 4.00 |
|  | Micronutrient fortified, maize-based food | 40 | 12.83 ± 11.83 | 8.97 | 40 | 22.53 ± 29.53 | 8.50 | 40 | 39.13 ± 69.11 | 19.33 |
|  | Highly processed maize-based foods | 40 | 1.28 ± 1.41 | 1.00 | 40 | 1.71 ± 2.11 | 1.00 | 40 | 2.18 ± 3.48 | 1.00 |
|  | Fats and oils | 40 | 0.85 ± 1.23 | 0.00 | 40 | 1.45 ± 2.09 | 1.00 | 39 | 1.09 ± 1.85 | 0.00 |
|  | Fruit | 40 | 0.45 ± 1.68 | 0.00 | 40 | 0.30 ± 0.79 | 0.00 | 40 | 0.33 ± 1.37 | 0.00 |
|  | Other vegetables | 40 | 33.58 ± 29.56 | 32.86 | 40 | 34.75 ± 23.38 | 31.43 | 40 | 44.36 ± 42.19 | 32.86 |
|  | Green and yellow vegetables | 39 | 7.04 ± 7.17 | 5.00 | 40 | 4.87 ± 3.76 | 4.00 | 40 | 5.97 ± 4.52 | 6.14 |
|  | Green leafy vegetables | 39 | 3.62 ± 4.88 | 2.25 | 40 | 5.39 ± 5.01 | 4.00 | 40 | 4.97 ± 11.73 | 2.00 |
|  | Fish | 40 | 3.43 ± 2.37 | 3.00 | 40 | 2.89 ± 2.51 | 2.25 | 40 | 4.87 ± 5.30 | 3.06 |
|  | Chicken | 40 | 1.52 ± 2.00 | 1.00 | 40 | 2.52 ± 1.79 | 2.00 | 40 | 1.22 ± 2.11 | 0.00 |
|  | Pork | 40 | 1.04 ± 1.42 | 0.65 | 40 | 1.02 ± 1.09 | 0.86 | 40 | 1.28 ± 2.09 | 0.54 |
|  | Beef food group | 40 | 1.15 ± 2.99 | 0.00 | 40 | 0.60 ± 2.10 | 0.00 | 40 | 1.13 ± 4.91 | 0.00 |
|  | Locally produced maize-based foods | 40 | 56.07 ± 38.27 | 44.50 | 39 | 57.39 ± 38.07 | 49.00 | 40 | 58.51 ± 34.31 | 55.25 |

|  |  | **San José Del Golfo** | | | **San José Pinula** | | | **San Juan Sacatepéquez** | | | |
| --- | --- | --- | --- | --- | --- | --- | --- | --- | --- | --- | --- |
|  |  | **N** | **Mean ± Std** | **Median** | **N** | **Mean ± Std** | **Median** | | **N** | **Mean ± Std** | **Median** |
| Summary statistics | Maize-based food consumption (g) | 43 | 2,280.44 ± 997.53 | 2,325.00 | 39 | 2,845.72 ± 1251.42 | 2,625.00 | | 39 | 4,180.64 ± 1341.77 | 4,155.00 |
|  | Percent of total food maize-based | 41 | 34.56 ± 12.74 | 34.19 | 39 | 40.28 ± 13.43 | 42.06 | | 39 | 51.91 ± 12.53 | 53.92 |
|  | Total grams consumed | 41 | 7,000.56 ± 3,396.64 | 5,892.75 | 39 | 7,307.95 ± 3,083.14 | 6,385.00 | | 39 | 8,209.06 ± 2,501.81 | 7,637.50 |
|  | Number of food types consumed | 45 | 20.69 ± 5.80 | 20.00 | 39 | 20.05 ± 4.74 | 19.00 | | 40 | 23.80 ± 6.36 | 24.00 |
|  | Number of maize-based foods | 45 | 4.60 ± 2.45 | 4.00 | 39 | 4.38 ± 2.17 | 4.00 | | 40 | 5.40 ± 2.46 | 5.00 |
| Food group (servings) | Eggs | 45 | 8.44 ± 7.28 | 8.00 | 39 | 7.69 ± 7.23 | 8.00 | | 40 | 9.08 ± 7.78 | 8.00 |
|  | Dairy | 45 | 1.86 ± 2.31 | 0.00 | 39 | 1.31 ± 2.34 | 0.00 | | 40 | 0.89 ± 2.12 | 0.00 |
|  | Bread | 45 | 12.93 ± 7.37 | 12.43 | 39 | 8.42 ± 5.82 | 7.00 | | 40 | 7.59 ± 5.96 | 6.00 |
|  | Rice | 44 | 1.82 ± 2.70 | 0.00 | 39 | 1.21 ± 2.20 | 0.00 | | 39 | 0.95 ± 1.69 | 0.00 |
|  | Grains | 45 | 27.60 ± 22.27 | 23.00 | 39 | 19.92 ± 20.20 | 14.00 | | 40 | 24.68 ± 20.12 | 20.50 |
|  | Nuts | 45 | 10.35 ± 13.53 | 5.98 | 39 | 13.38 ± 16.60 | 8.00 | | 40 | 8.72 ± 6.54 | 8.00 |
|  | Micronutrient fortified, maize-based food | 45 | 19.44 ± 26.53 | 16.00 | 39 | 34.12 ± 70.90 | 17.67 | | 40 | 17.13 ± 12.07 | 16.00 |
|  | Highly processed maize-based foods | 45 | 2.16 ± 2.36 | 1.00 | 39 | 1.35 ± 2.12 | 1.00 | | 40 | 2.90 ± 3.77 | 2.00 |
|  | Fats and oils | 44 | 1.19 ± 1.97 | 0.00 | 39 | 0.75 ± 2.25 | 0.00 | | 40 | 0.93 ± 2.42 | 0.00 |
|  | Fruit | 44 | 0.36 ± 0.84 | 0.00 | 39 | 0.36 ± 1.25 | 0.00 | | 40 | 0.63 ± 1.61 | 0.00 |
|  | Other vegetables | 45 | 52.55 ± 36.03 | 45.00 | 39 | 54.04 ± 54.82 | 32.86 | | 40 | 54.00 ± 40.34 | 40.71 |
|  | Green and yellow vegetables | 45 | 15.23 ± 19.64 | 7.00 | 39 | 5.32 ± 6.15 | 3.75 | | 40 | 4.20 ± 3.52 | 3.14 |
|  | Green leafy vegetables | 45 | 3.90 ± 5.36 | 3.00 | 39 | 7.56 ± 10.34 | 4.00 | | 40 | 7.72 ± 9.49 | 4.56 |
|  | Fish | 45 | 4.53 ± 6.42 | 3.00 | 39 | 3.13 ± 2.86 | 2.25 | | 40 | 2.77 ± 2.46 | 2.13 |
|  | Chicken | 45 | 1.81 ± 2.10 | 1.00 | 39 | 1.65 ± 1.79 | 1.00 | | 40 | 2.85 ± 3.74 | 1.15 |
|  | Pork | 43 | 1.94 ± 2.41 | 1.87 | 39 | 0.91 ± 1.49 | 0.00 | | 40 | 2.10 ± 1.75 | 1.51 |
|  | Beef food group | 45 | 0.04 ± 0.30 | 0.00 | 39 | 1.13 ± 4.18 | 0.00 | | 40 | 1.31 ± 3.20 | 0.00 |
|  | Locally produced maize-based foods | 45 | 48.06 ± 22.95 | 48.00 | 39 | 64.95 ± 31.57 | 63.00 | | 40 | 95.30 ± 34.38 | 97.00 |

|  |  | **San Miguel Petapa** | | | **San Pedro Ayampuc** | | | **San Pedro Sacatepéquez** | | |
| --- | --- | --- | --- | --- | --- | --- | --- | --- | --- | --- |
|  |  | **N** | **Mean ± Std** | **Median** | **N** | **Mean ± Std** | **Median** | **N** | **Mean ± Std** | **Median** |
| Summary statistics | Maize-based food consumption (g) | 45 | 2,763.91 ± 1436.27 | 2,640.00 | 44 | 2,687.61 ± 1,225.53 | 2,520.00 | 45 | 3,520.58 ± 1,496.65 | 3,432.00 |
|  | Percent of total food maize-based | 45 | 37.15 ± 16.96 | 36.47 | 39 | 38.02 ± 15.20 | 38.76 | 45 | 48.56 ± 14.14 | 46.85 |
|  | Total grams consumed | 45 | 7,434.84 ± 2,676.27 | 6,802.50 | 39 | 7,704.60 ± 3,421.74 | 7,033.19 | 45 | 7,288.02 ± 2,188.55 | 6,951.00 |
|  | Number of food types consumed | 45 | 21.11 ± 5.26 | 20.00 | 45 | 21.04 ± 7.66 | 20.00 | 45 | 21.98 ± 5.72 | 22.00 |
|  | Number of maize-based foods | 45 | 4.31 ± 1.78 | 4.00 | 45 | 4.69 ± 2.63 | 4.00 | 45 | 5.29 ± 2.13 | 5.00 |
| Food group (servings) | Eggs | 45 | 12.58 ± 10.71 | 8.00 | 45 | 8.74 ± 8.32 | 8.00 | 45 | 7.08 ± 6.13 | 4.00 |
|  | Dairy | 45 | 2.53 ± 4.68 | 0.00 | 45 | 1.82 ± 3.63 | 0.00 | 45 | 1.24 ± 2.34 | 0.00 |
|  | Bread | 45 | 9.92 ± 8.63 | 7.00 | 44 | 10.68 ± 9.22 | 7.86 | 45 | 9.56 ± 7.56 | 7.00 |
|  | Rice | 45 | 1.60 ± 2.46 | 0.00 | 45 | 1.33 ± 2.71 | 0.00 | 45 | 1.27 ± 2.30 | 0.00 |
|  | Grains | 45 | 28.62 ± 25.90 | 22.00 | 45 | 26.60 ± 24.02 | 25.00 | 45 | 30.16 ± 20.80 | 28.00 |
|  | Nuts | 45 | 11.06 ± 11.23 | 8.00 | 45 | 8.55 ± 14.53 | 2.00 | 45 | 8.60 ± 8.07 | 8.00 |
|  | Micronutrient fortified, maize-based food | 45 | 21.99 ± 28.42 | 16.00 | 45 | 31.17 ± 46.63 | 16.00 | 45 | 19.28 ± 29.62 | 11.97 |
|  | Highly processed maize-based foods | 45 | 2.61 ± 4.65 | 1.00 | 45 | 2.24 ± 3.74 | 1.00 | 45 | 2.36 ± 3.66 | 1.00 |
|  | Fats and oils | 45 | 0.83 ± 1.33 | 0.00 | 45 | 0.98 ± 1.96 | 0.00 | 45 | 1.02 ± 1.45 | 1.00 |
|  | Fruit | 45 | 0.44 ± 1.71 | 0.00 | 45 | 0.22 ± 0.60 | 0.00 | 45 | 0.60 ± 1.45 | 0.00 |
|  | Other vegetables | 45 | 44.85 ± 28.76 | 32.86 | 45 | 41.43 ± 36.13 | 30.00 | 45 | 42.41 ± 28.69 | 32.86 |
|  | Green and yellow vegetables | 45 | 8.18 ± 8.63 | 7.00 | 42 | 19.09 ± 66.97 | 7.00 | 45 | 6.60 ± 10.50 | 4.00 |
|  | Green leafy vegetables | 45 | 6.79 ± 8.04 | 4.00 | 44 | 7.03 ± 16.95 | 3.38 | 45 | 4.99 ± 6.24 | 3.00 |
|  | Fish | 45 | 3.39 ± 2.53 | 3.00 | 44 | 4.05 ± 3.01 | 3.38 | 45 | 2.78 ± 1.92 | 2.50 |
|  | Chicken | 45 | 2.83 ± 3.03 | 1.88 | 45 | 1.60 ± 1.91 | 1.00 | 45 | 2.23 ± 2.37 | 1.50 |
|  | Pork | 45 | 2.05 ± 3.74 | 0.58 | 45 | 2.12 ± 2.97 | 1.15 | 45 | 1.31 ± 2.22 | 1.00 |
|  | Beef food group | 45 | 2.25 ± 4.34 | 0.00 | 45 | 1.63 ± 3.79 | 0.00 | 45 | 1.62 ± 5.54 | 0.00 |
|  | Locally produced maize-based foods | 45 | 61.56 ± 34.74 | 58.00 | 44 | 58.63 ± 29.37 | 59.00 | 45 | 81.45 ± 36.33 | 80.25 |

|  |  | **San Raymundo** | | | **Santa Catarina Pinula** | | | **Villa Canales** | | |
| --- | --- | --- | --- | --- | --- | --- | --- | --- | --- | --- |
|  |  | **N** | **Mean ± Std** | **Median** | **N** | **Mean ± Std** | **Median** | **N** | **Mean ± Std** | **Median** |
| Summary statistics | Maize-based food consumption (g) | 39 | 3,089.76 ± 1,660.08 | 2,890.00 | 43 | 2,483.53 ± 1,595.09 | 2,295.00 | 45 | 2,983.89 ± 1,596.86 | 2,791.00 |
|  | Percent of total food maize-based | 39 | 44.30 ± 13.31 | 43.73 | 43 | 33.35 ± 13.93 | 31.36 | 44 | 41.79 ± 15.47 | 40.07 |
|  | Total grams consumed | 39 | 6,889.76 ± 2,739.69 | 6,823.50 | 43 | 7,222.03 ± 3,561.07 | 6,661.38 | 44 | 7,014.72 ± 2,418.35 | 7,069.88 |
|  | Number of food types consumed | 40 | 22.30 ± 6.39 | 21.50 | 44 | 20.95 ± 5.81 | 20.00 | 45 | 21.76 ± 6.07 | 22.00 |
|  | Number of maize-based foods | 40 | 5.55 ± 2.43 | 5.00 | 44 | 4.91 ± 2.23 | 5.00 | 45 | 5.11 ± 2.41 | 5.00 |
| Food group (servings) | Eggs | 40 | 7.53 ± 5.76 | 6.00 | 44 | 9.95 ± 13.26 | 6.00 | 45 | 7.88 ± 7.28 | 4.00 |
|  | Dairy | 40 | 0.85 ± 1.82 | 0.00 | 44 | 2.70 ± 7.70 | 0.00 | 45 | 1.30 ± 2.66 | 0.00 |
|  | Bread | 40 | 11.31 ± 13.77 | 7.67 | 44 | 10.80 ± 7.74 | 8.50 | 44 | 10.71 ± 6.32 | 9.96 |
|  | Rice | 40 | 1.46 ± 2.57 | 0.00 | 44 | 1.57 ± 2.80 | 0.50 | 45 | 1.91 ± 2.92 | 0.00 |
|  | Grains | 40 | 17.58 ± 12.95 | 14.00 | 44 | 31.52 ± 35.38 | 24.00 | 44 | 20.82 ± 14.81 | 18.00 |
|  | Nuts | 40 | 8.20 ± 11.89 | 4.99 | 44 | 16.22 ± 23.45 | 8.00 | 45 | 14.53 ± 20.01 | 8.00 |
|  | Micronutrient fortified, maize-based food | 40 | 16.46 ± 27.86 | 8.00 | 44 | 19.62 ± 24.88 | 14.09 | 45 | 17.28 ± 15.11 | 16.00 |
|  | Highly processed maize-based foods | 40 | 2.23 ± 2.29 | 2.00 | 43 | 3.26 ± 3.89 | 1.00 | 45 | 1.46 ± 2.27 | 1.00 |
|  | Fats and oils | 40 | 0.75 ± 1.53 | 0.00 | 44 | 2.32 ± 6.67 | 0.50 | 45 | 0.96 ± 1.47 | 0.08 |
|  | Fruit | 39 | 0.77 ± 1.65 | 0.00 | 44 | 0.39 ± 0.99 | 0.00 | 45 | 1.38 ± 4.64 | 0.00 |
|  | Other vegetables | 40 | 35.39 ± 27.57 | 30.00 | 44 | 54.41 ± 72.95 | 32.86 | 45 | 34.93 ± 20.47 | 32.86 |
|  | Green and yellow vegetables | 40 | 5.70 ± 7.10 | 3.25 | 44 | 9.85 ± 11.88 | 6.00 | 45 | 6.16 ± 9.37 | 4.00 |
|  | Green leafy vegetables | 40 | 7.26 ± 6.63 | 6.56 | 44 | 6.48 ± 8.12 | 2.88 | 45 | 5.26 ± 6.32 | 3.00 |
|  | Fish | 40 | 5.86 ± 3.92 | 4.94 | 44 | 3.82 ± 3.71 | 3.00 | 45 | 3.52 ± 3.13 | 2.50 |
|  | Chicken | 40 | 2.09 ± 3.34 | 1.00 | 44 | 2.94 ± 4.14 | 1.71 | 45 | 1.78 ± 1.78 | 1.00 |
|  | Pork | 40 | 1.59 ± 2.46 | 1.00 | 44 | 1.26 ± 1.19 | 1.08 | 45 | 1.74 ± 2.80 | 1.00 |
|  | Beef food group | 40 | 1.16 ± 2.34 | 0.00 | 44 | 0.21 ± 0.96 | 0.00 | 45 | 1.80 ± 5.38 | 0.00 |
|  | Locally produced maize-based foods | 40 | 69.75 ± 41.12 | 64.00 | 43 | 49.79 ± 35.41 | 45.00 | 45 | 65.79 ± 38.77 | 64.00 |

|  |  | **Villa Nueva** | | | **P-value*** |
| --- | --- | --- | --- | --- | --- |
|  |  | **N** | **Mean ± Std** | **Median** |  |
| Summary statistics | Maize-based food consumption (g) | 45 | 2,802.59 ± 1,832.48 | 2705.00 | <0.001 |
|  | Percent of total food maize-based | 45 | 39.51 ± 14.48 | 41.35 | <0.001 |
|  | Total grams consumed | 45 | 6,843.65 ± 3,153.48 | 6146.00 | <0.001 |
|  | Number of food types consumed | 45 | 20.11 ± 5.09 | 21.00 | 0.019 |
|  | Number of maize-based foods | 45 | 4.51 ± 1.89 | 5.00 | 0.204 |
| Food group (servings) | Eggs | 45 | 8.18 ± 8.38 | 8.00 | <0.001 |
|  | Dairy | 45 | 1.18 ± 1.87 | 0.00 | 0.496 |
|  | Bread | 45 | 10.10 ± 8.62 | 8.00 | <0.001 |
|  | Rice | 45 | 1.07 ± 1.94 | 0.00 | 0.003 |
|  | Grains | 45 | 31.96 ± 32.42 | 21.00 | <0.001 |
|  | Nuts | 45 | 8.93 ± 9.75 | 8.00 | 0.020 |
|  | Micronutrient fortified, maize-based food | 45 | 19.39 ± 26.46 | 11.97 | 0.151 |
|  | Highly processed maize-based foods | 45 | 1.33 ± 1.65 | 1.00 | <0.001 |
|  | Fats and oils | 45 | 1.30 ± 2.29 | 1.00 | 0.591 |
|  | Fruit | 45 | 0.42 ± 1.08 | 0.00 | 0.022 |
|  | Other vegetables | 45 | 28.69 ± 23.45 | 30.00 | <0.001 |
|  | Green and yellow vegetables | 45 | 5.74 ± 4.61 | 4.03 | <0.001 |
|  | Green leafy vegetables | 45 | 5.78 ± 5.56 | 4.00 | 0.012 |
|  | Fish | 45 | 2.74 ± 2.13 | 2.50 | <0.001 |
|  | Chicken | 45 | 1.47 ± 1.80 | 1.00 | <0.001 |
|  | Pork | 45 | 1.73 ± 1.88 | 1.15 | <0.001 |
|  | Beef food group | 45 | 1.78 ± 4.64 | 0.00 | 0.012 |
|  | Locally produced maize-based foods | 45 | 63.40 ± 45.11 | 60.00 | <0.001 |

*p-value calculated using chi-square test
